# Empirical evaluation of analytic validity of polygenic scores

**DOI:** 10.1186/s13073-026-01654-6

**Published:** 2026-04-23

**Authors:** Tian Lin, Jian Zeng, Scott D. Gordon, Leanne Wallace, Laura Ziser, Sonia Shah, Oliver Pain, Ilja M. Nolte, Harold Snieder, Lifelines Cohort Study, Raul Aguirre-Gamboa, Patrick Deelen, Lude Franke, Jan A Kuivenhoven, Esteban A Lopera Maya, Serena Sanna, Morris A Swertz, Judith M Vonk, Cisca Wijmenga, Paul A. James, Nicholas G. Martin, Peter M. Visscher, Eric Lee, Loic Yengo, Anjali K. Henders, Naomi R. Wray

**Affiliations:** 1 Institute for Molecular Bioscience, The University of Queensland, Brisbane, Australia; 2 Brain and Mental Health Program, QIMR Berghofer Medical Research Institute, Brisbane, Australia; 3 Department of Basic and Clinical Neuroscience, Institute of Psychiatry, Psychology and Neuroscience, Maurice Wohl Clinical Neuroscience Institute, King’s College London, London, UK; 4 Department of Epidemiology, University of Groningen, University Medical Center Groningen, Groningen, The Netherlands; 5 Peter MacCallum Cancer Centre, Melbourne, Australia; 6 Nuffield Department of Population Health, University of Oxford, Oxford, UK; 7 Preciselee, Sydney, NSW 2065 Australia; 8 Department of Psychiatry, University of Oxford, Oxford, UK

**Keywords:** Polygenic scores, Polygenic risk, Technical replication, Reproducibility

## Abstract

**Background:**

Polygenic scores (PGSs) are weighted sum scores of trait-associated alleles from up to millions of SNPs. As PGS research pivots to translation into health care settings a key issue for laboratories providing PGS is demonstration of analytical validity of PGS.

**Methods:**

We report data from 6 individuals who have been genotyped multiple times using the same and different technologies. These data were generated as part of standard experimental design for quality control purposes in two research settings over many studies and over many years. Using this opportunistic design of technical variability, we provide an empirical evaluation of technical reproducibility of PGS from 115 traits of different genetic architectures.

**Results:**

Given a predefined set of SNP weights variability in PGS can reflect only SNP missingness or incorrect genotype call. We find very high reproducibility of SNP genotypes. In particular, the technical reproducibility of PGS generated from the same array technology and processed through the same quality control and imputation pipeline is very high. However, impact of missing SNPs varies between traits depending on the SNP’s weight for a trait. We provide a PGS quality score statistic (PGS:QS) that can be reported for each trait-specific score for an individual, to provide a quantitative assessment of the proportion of variation of the score that is captured by the SNPs genotyped/imputed for the individual. We provide an algorithm (PGS-impute) that updates the SNP weights of the scoring algorithm to the SNPs available for an individual, improving PGS accuracy.

**Conclusions:**

While validity of directly measured genotypes (whether from microarray or whole genome sequencing) is well-established, objective approaches to evaluate analytical reproducibility of PGS post-genotyping pipeline have been lacking. Here, we provide empirical data and an analysis framework which can be used by PGS providers to support understanding of analytical reproducibility and robustness.

**Supplementary Information:**

The online version contains supplementary material available at 10.1186/s13073-026-01654-6.

## Background

Polygenic scores (PGS) provide estimates of the genetic value of a trait for an individual. In the context of disease traits, the PGS represent the estimated genetic risk of the disease for an individual, also known as polygenic risk scores or PRS. Genetic factors only represent part of the variation in liability of risk of disease, and PGS only capture a fraction of this risk [1]. Hence, PGS are not diagnostic tests, but are biomarkers that are likely to be combined with other risk factors in risk prediction or disease stratification algorithms. PGS are now extensively used in research and are increasingly being evaluated in healthcare settings. While national and international regulatory frameworks govern clinical tests, accreditation for PGS is breaking new ground. For example, consider the Australian regulatory approach, which is likely to be internationally representative, at least of countries with state-funded health systems. The Australian National Pathology Accreditation Advisory Council (NPAAC) considers PGS to be In Vitro Diagnostic Medical Devices, categorising them as Multivariate Index Assays (MIAs) [2]. An MIA is defined as a test which (a) combines the values of multiple variables using an interpretation function to yield a final, patient-specific result and (b) provides a result whose derivation is non-transparent and cannot be independently derived or verified by the end user. To date, a PGS test has yet to be registered with the Medical Services Advisory Committee (MSAC) or the Therapeutic Goods Administration (TGA), which are required for a test to be government funded. The guidelines provided in the July 2021 MSAC report [3] for a gene expression MIA provide pointers for registration expectations. These include that the test should be performed in an Australian laboratory accredited by the National Association of Testing Authorities (NATA) according to NPAAC standards. To date, two companies have successfully achieved NATA accreditation for PGS.

The most important part of seeking accreditation of PGS will be demonstration of the utility of the test in its contribution to trait or disease prediction. This would include demonstrated accuracy of the predictor both within and across ancestries [4], uncertainty of PGS for an individual given uncertainty in SNP effect estimates [5] and calibration of the predictor [6]. Given these critical but “transparent” features, accreditation is sought for a defined SNP set with assigned weights for one SNP allele, contrasting the zero weight for the other allele. Given this SNP list, a “non-transparent” component of PGS is that they can use genotypes of millions of DNA variants (mostly single nucleotide polymorphisms, SNPs), of which only a fraction (e.g., less than 10%) might have been directly measured, depending on genotyping technology. Therefore, from the standpoint of test accreditation, generation of PGS has unquantified technical uncertainties. Here, using the language of the ACCE (Analytic validity, Clinical validity, Clinical utility, Ethical, legal and social implications) framework for genetic tests [7] we focus on analytic validity and reproducibility of PGS and provide empirical data to help support understanding of technical variability.

PGS are calculated as weighted counts of trait-associated alleles. Hence, PGS can be reproducibly calculated from the genetic data stored for an individual when calculated from a predefined list of SNPs and score weights linked to a specified allele for a given trait. Unambiguous reproducibility can be achieved if any PGS reported can be linked to a registered set of SNP weights (recognising that these may be proprietary) through a PGS numbering system, such as the persistent identifier used in the PGS catalog [8]. Therefore, the only way that technical variability (across technical replication of genome-wide genotyping) can enter the calculation is when the genotype for an individual is incorrect or missing. Incorrect or missing genotypes could occur either in direct genotyping/sequencing or through the imputation process which is applied to genotyping array or low coverage whole genome sequence (lcWGS) data [9]. Imputation exploits the correlation structure (i.e., linkage disequilibrium, LD) in the genome to provide genotypes of SNPs (and other DNA variants) that are not directly measured but whose values can be inferred from reference data. The reference data comprise thousands of individuals who have been genotyped through whole genome sequencing. Their diploid SNP genotypes across all chromosomes are phased into haplotypes (chromosome segments of SNP allele combinations, which are present in a proportion of chromosomes in the population). Importantly, the reference data should include individuals drawn from many ancestries, because even individuals self-reported to have homogeneous genetic ancestry (e.g., 4 grandparents born in the same worldwide geographic region) may have distant ancestors from other ancestral groups resulting in haplotype segments from those ancestors. Only SNPs found in the reference data can be imputed, hence larger reference data sets represent more haplotypes although there are diminishing returns in new SNP discovery with increasing sample size [10]. It has long been recognised (see for example Fig. 3 of Yang et al. [11] that, in general, common DNA variants can be imputed accurately, but less common variants (which by definition are less likely to be correlated with the genotyped variants [12]) are not. Since PGS SNP weights are derived from genome-wide same genetic association study (GWAS) results, rare variants (< 0.5%) do not feature in current PGS SNP lists. Hence, best-guess imputed genotypes are accurate for common SNPs, and so there is little gain from using dosage genotypes in PGS [13, 14]. Correlation matrices (i.e., LD r^2^ matrices) for SNPs are available to researchers (e.g., GCTB downloads, see URL) for different ancestry populations which have different correlation structures reflecting the evolutionary histories of populations over many generations (most notably population size, but also selection, migration and population mixing).

**Fig. 1:**
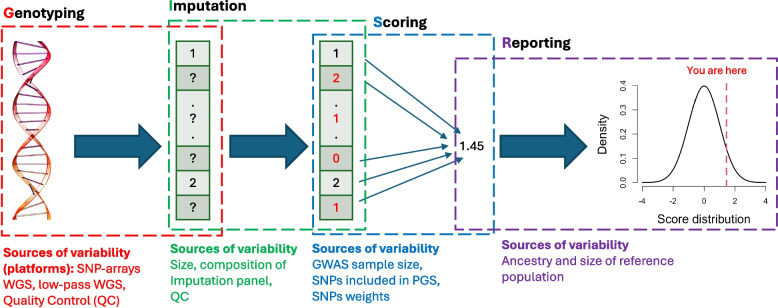
Sources of variability in generation of PGS

Figure 1 summarises sources of variability in generation of PGS: technical variability in genotyping technology (G), variability because of the choice of reference panel used for imputation (I), variability in the PGS scoring algorithm (S) and variability in the reporting of PGS (R). Here, we evaluate technical variability based on the G and I steps and consider the benchmarking sample in the R step. The S step is the most researched; methods to optimise SNP weights is an active area of research [15–18]. PGS will continue to improve over time, both from ever-increasing GWAS sample sizes and new methodologies to optimise estimates of SNP weights. These improvements, by definition, will lead to re-ranking of individuals which could be difficult to manage in clinical practice [19]. Hence, the S step is clearly the driving force of PGS implementation, but here we assume the SNP weights for each trait have been selected and locked-in in order to focus on the technical reproducibility of PGS which is relatively under-researched. We hope empirical data can inform general understanding of what can be expected in real-world settings. We consider the S step through study of PGS for many traits which have different architectures.

Here, we present an opportunistic study in which we use data accumulated in two independent research groups over many years, providing repeated genotyping of 6 individuals across different platforms and different genotyping providers. Although not a planned experimental design, these data provide useful empirical evaluation of technical variation impacting PGS. We explore the impact of technical variability across PGS of 83 quantitative and 32 disease traits. We show that PGS are generally very robust to technical variability, consistent with previous studies of technical reproducibility of array genotypes generated across sites [20], and comparison of array and WGS [21]. We show that repeated array genotyping of DNA from an individual followed by processing through a quality control (QC) pipeline with imputation to the same reference panel provides highly consistent imputed genotypes and hence PGS. However, we show that choice of reference panels leads to differences in SNP missingness (reflecting SNPs present in the reference data, and hence the missingness reproduces over technical replicates). Across the 115 traits studied we show that the missing SNPs mostly have little impact on the PGS, except when the missing SNPs happen to be SNPs with large effect for a particular trait. We propose a PGS quality score (PGS:QS) to be reported alongside each PGS calculated for an individual and for each trait that quantifies the impact of missing SNPs missing in their individual genomic profile. We provide a method (PGS-impute) to re-calculate SNP weights based on the SNPs available in an individual sample which recovers the PGS estimate. We provide a checklist of factors to consider when evaluating analytic validity of a PGS. Our data and framework provide a pathway to support PGS providers and regulators to be confident that technical reproducibility can be achieved.

## Methods

### Samples and genotyping

This study uses genome-wide genotype data generated on DNA purchased as lymphoblastoid cell lines of the Centre d'Etude du Polymorphism Humain (CEPH) samples (north-west European ancestry) from Coriell Institute of Medical Research. Data used have been generated by two independent groups in Brisbane, Australia: at the Human Studies Unit (HSU) of the University of Queensland and QIMR Berghofer Medical Research Institute (QIMRB). DNA from a cell-line individual has been included on every genotyping array and in every sequencing study (a QC in experimental design, for example to quickly identify faulty batches of arrays). Cell lines provide high quality DNA which well represents the DNA quality expected in NATA accreditation for generation of genotype data. Two CEPH individuals (NA06997, NA07029) were genotyped across multiple SNP arrays as well as lcWGS (~ 1X) (Table 1; Additional file 1: Table S1). High coverage (30X) WGS data for these individuals were available from the International Genome Sample Resource (see URL). Four CEPH individuals were genotyped by QIMRB multiple times on the same array (Illumina GSAMD-24v1-0_20011747 or GSA + MDv1) NA06990 (genotyped 35 times (*N* = 35)), NA07023 (*N* = 37), NA07057 (*N* = 34), NA10861 (*N* = 106). Of these, WGS was only available for NA10861. lcWGS and Illumina Global Screening array (GSA) data from the same 46 HSU collected samples are also used.

**Table 1:** Genome-wide genotyping platforms for CEPH individuals NA06997 and NA0702

Array^a^	Array Short Name	Source	Genotyping Lab	#SNPs^b^	NA06997	NA07029
Illumina GSA v3b with MD content	IGSAv3 + MD	HSU	HSU	487,748	29	21
Illumina GSA v3 with MD + custom content	IGSAv3 + MD + UQ	HSU	HSU	488,479	32	20
Illumina GSA v2 with MD content	IGSAv2 + MD	HSU	HSU	499,664	3	3
Axiom SARS-CoV-2	ASARS-COV2	HSU	Ram	500,112	1	1
Illumina Human610-Quadv1_B	I610Qv1	QIMRB	CIDR	528,889	0	3
Illumina Human660W-Quad_v1_C	I660Wv1	QIMRB	CIDR	510,297	0	1
WGS 30X read depth	WGS	IGSR	NYGC	N/A	1	1
WGS 1X read depth	lcWGS	HSU	AGRF	N/A	1	0

*GSA* Global screening array, *MD* Major disease, *HSU* University of Queensland Human Studies Unit, Australia, *QIMRB* QIMR Berghofer Institute of Medical Research, Australia, *Ram* Ramaciotti Institute, Australia, *NCI* National Cancer Institute, USA, *CIDR* Center for Inherited Disease Research, USA, *1000G* 1000Genomes, *AGRF* Australian Genome Research Facility, Australia

^a^See Supplementary Tables 1 and 2 for more details

^b^Directly measured SNPs passing QC and used for imputation

### Imputation

All data were processed through in-house QC pipelines built with reference to best practice guidelines [22] (see Additional file 2: Supplementary Note), developed independently at HSU and QIMRB. The HSU pipeline used the Haplotype Reference Consortium (HRC) Release 1.1 imputation panel [23] (*N* = 27,165 individuals) downloaded to our own high performance computing server. The lcWGS data were also imputed to this version of the reference. The QIMRB samples were imputed with both HRC imputation panel (Michigan Imputation server, *N* = 32,488 individuals) and TOPMed r3 panel [24] (TOPMed imputation server, *N* = 53,831). Given that both Chen et al. [13] and Privé et al. [14] found that the variability in PGS attributable to imputation (including use of best guess genotypes over dosage scores) is minor, we do not investigate imputation methods and use best guess genotypes.

### Scoring

European ancestry GWAS summary statistics for 32 binary traits and 83 quantitative traits (including 16 fatty acids [25], 10 proteins [26] and 34 blood/urine biomarkers [27]) (Additional file 1: Table S2; Additional file 2: Fig S1) are used in this study. They were processed into a consistent format (see Github code) and through the SBayesRC [17] default QC criteria implemented in the GCTB software. For most traits the SBayesRC default European ancestry high density LD matrix of 7,356,518 SNPs (minor allele frequency > 0.01) could be used to represent the correlation between the marginal SNP effects provided in the GWAS data. However, for 4 traits the HapMap3 LD reference of 1.2 M SNPs was used owing to low coverage of SNPs in the original GWAS data. We chose SBayesRC to generate SNP weights from GWAS data because it is one of the latest generations of PGS methods, providing optimised SNP weights given the LD structure between SNPs. A standard GWAS analysis calculates marginal SNP effect sizes (e.g., SNPs correlated with a causal SNP of large effect will all have estimated large effects) whereas the Bayesian multiple regression estimates are joint effect sizes (e.g., SNPs correlated with large estimated marginal effect size will have Bayesian effects that sum to reflect the contribution of the locus). The SBayesRC method provides a general and flexible framework modelling SNP effects to be drawn from a mixture of normal distributions with difference variances. The proportions of SNPs allocated to each distribution is estimated from the data, and in this way, any genetic architecture is modelled. The flexible framework includes SNP annotations, for example functional annotations (such as enhancer, promoter, conserved, coding) allowing optimised estimation of SNP effects. Many published studies (e.g., [28–30] have compared PGS methods and choice of a single method for generating SNP weights will not impact the conclusions from this study.

For each individual *m* their PGS for trait* j* was calculated as $PG{S}_{m,j}= \sum_{i=1}^{n}{x}_{i}{\widehat{\beta }}_{i,j}$, where ${x}_{i}$ is the number of alleles (0,1,or 2) at SNP *i* of the allele with SBayesRC SNP weight ${\widehat{\beta }}_{i,j}$ for the trait. The sum is over $n$ = 7.4 M (or 1.2 M) SNPs with SBayesRC weights.

### Reporting

PGS were benchmarked against two independent cohorts of inferred European ancestry. For most comparisons, PGS were standardised based on the mean and standard deviation of PGS calculated in a subset of the Lifelines cohort (*N* = 36,237) (participants recruited in the northern provinces of the Netherlands, see Additional file 2: Supplementary Note) genotyped on the Thermofisher Axiom_HGCoV2_1.r3 array [31, 32]. For evaluation of PGS in the context of benchmark samples, we also used an Australian cohort (*N* = 6,587) which comprised individuals genotyped at HSU (Illumina GSA). Both cohorts were imputed to the HRC 1.1. To exclude the impact of size of reference sample, we also used a benchmarked sample of *N* = 6,587 randomly down-sampled from the Lifelines cohort.

### PGS:Quality Score (PGS:QS)

The SBayesRC output file includes estimates (posterior means) of effect sizes (${\widehat{\beta }}_{i,j}$) of each SNP *i*, for the PGS trait *j*, and also an estimate of the variance explained by each SNP for the trait (${\widehat{V}}_{i,j}$) (Additional file 1: Table S2). The SBayesRC ${\widehat{\beta }}_{i,j}$ are joint effect sizes, hence the variances explained by SNPs are independent given the LD between causal variants and can be summed for each trait to give the total estimated variance ($\sum {\widehat{V}}_{i,j}={\widehat{V}}_{\cdot ,j}$) captured by the PGS in the SNP list. We observe that the ${\widehat{V}}_{\cdot ,j}$ value is, in general, consistent with the SNP-based heritability estimate for the trait *j* (Additional file 2: Fig S2), with the difference likely reflecting LD between causal variants. The technical variation of a PGS can come from SNP missingness. For genotyping instance* k*, and trait* j*, the summed variances across non-missing SNPs can be calculated (${\widehat{V}}_{\cdot ,j[k]}$). The PGS quality score (PGS:QS) is then calculated as $PGS:Q{S}_{j[k]}= \frac{{\widehat{V}}_{\cdot,\ j[k]}}{{\widehat{V}}_{\cdot,\ j}}$. This is calculated for each PGS trait as the missing SNPs will impact differently for each trait.

### PGS-impute

When SNPs in the PGS list are missing from the genotype data of a sample, then SNP weights on the full-set of SNPs (denoted here as SNP-set 1, $\widehat{{{\boldsymbol{\upbeta}}}_{1}}$) can be updated to provide weights only for the SNP genotypes available (SNP-set 2, $\widehat{{{\boldsymbol{\upbeta}}}_{2})}$ as $\widehat{{{\boldsymbol{\upbeta}}}_{2}}$**=**${\mathbf{D}}_{2}^{-1}{\mathbf{R}}_{22}^{-1}{\mathbf{R}}_{21}{\mathbf{D}}_{1}\widehat{{{\boldsymbol{\upbeta}}}_{1}}$ where $\mathbf{R}$ are LD matrices linking the SNPs denoted by their subscripts, **D** are diagonal matrices of the square root of SNP heterozygosity ($\sqrt{2p(1-p)}$ with $p$ being allele frequency) (see Additional file 2: Supplementary Note for the derivation).

## Results

### Genotyping and scoring

**Fig. 2:**
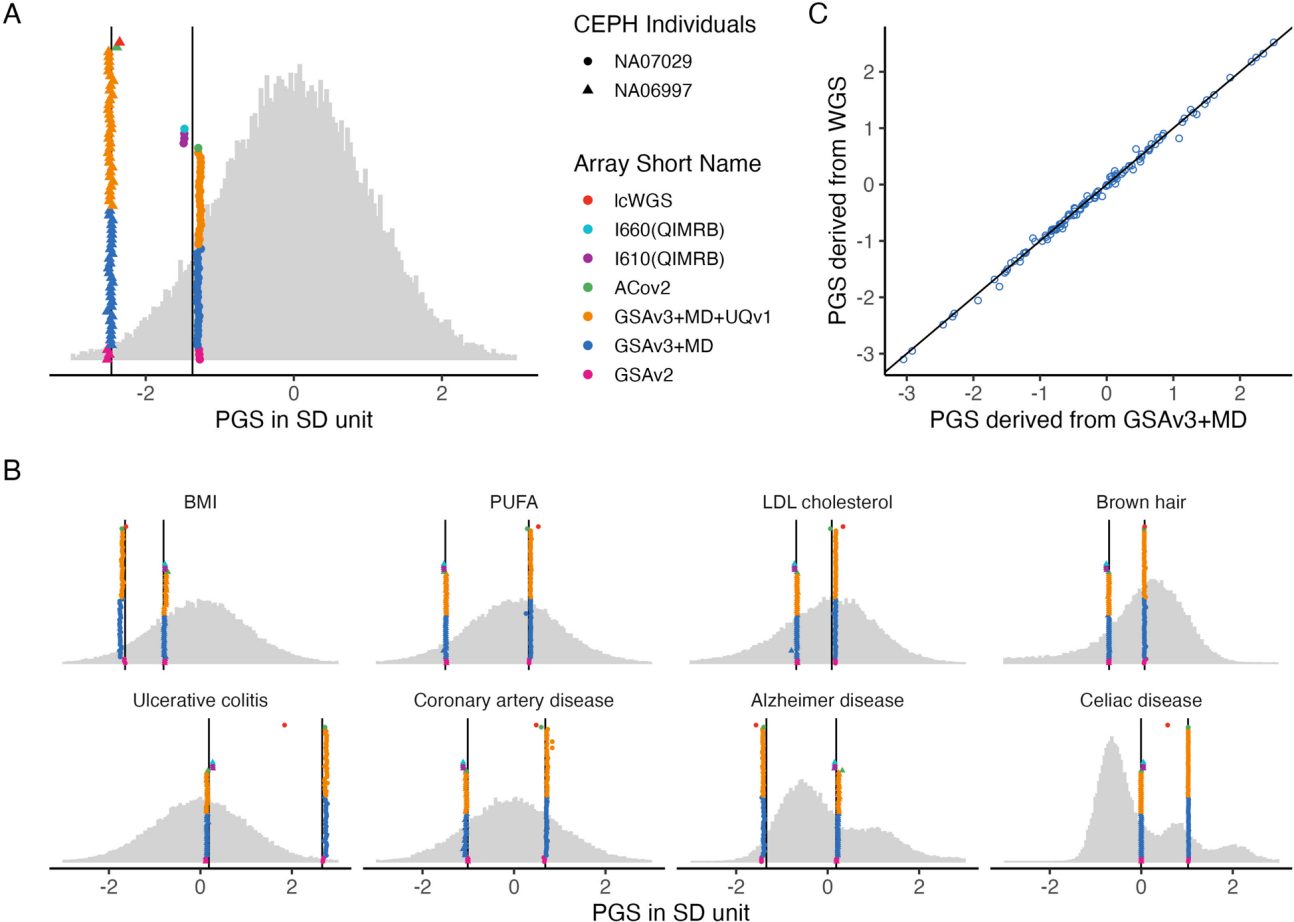
Genotyping & Scoring: PGS for two CEPH individuals genotyped multiple times**. A** PGS for height for CEPH individuals NA07029 (circles) and NA069977 (triangles) genotyped on different platforms (colours). **B** The majority of trait PGS show good agreement between PGS regardless on the genotyping platform (see Additional file 2: Fig S3) including for traits which have variants of large effects which generate non-normal population distributions of PGS. **C** PGS for 115 traits for a genotyping instance (x-axis) compared to the PGS for the same individual calculated from WGS data. For A) and B) The grey distributions of standardised PGS scores are from 36,237 benchmark samples of European inferred ancestry from the Lifelines cohort. The black vertical lines are PGS calculated from 30× WGS. BMI: body mass index; PUFA: Polyunsaturated fatty acids (see definition in Additional file 1: Table S3); LDL: low density lipoprotein

Two CEPH samples (NA06997, NA07029) were genotyped 64 and 44 times respectively on three similar Illumina arrays (Table 1; Additional file 1: Table S1) as part of over 75 genotyping batches at HSU. Of the 7,356,518 SNPs with SBayesRC SNP weights all but 52 (0.007%) were present in the HRC imputed data (Additional file 1: Table S2). PGS were calculated for each genotyping instance for each of 115 traits using SNP weights calculated from SBayesRC. For example, Fig. 2A shows the grey distribution of PGS scores for height for the 36,237 unrelated European ancestry-inferred individuals from Lifelines cohort used as benchmark samples to standardise the PGS. Each circle or triangle represent a PGS calculated from a genotyping event for NA07029 and NA06997, respectively. The colours represent different genotyping platforms. From bottom up, the pink, blue and orange symbols represent the PGS from the three versions of the Illumina GSA used at HSU. The green symbol (NA07029 only) is for a single genotyping event on the Axiom SARS-COV2 array. NA06997 was also genotyped on older Illumina arrays at QIMRB and processed through their independent in-house QC pipeline to generate PGS (purple and light blue symbols). The red symbol is for the PGS derived from lcWGS (NA06997 only). The black vertical lines are PGS calculated from WGS data. Figures similar to Fig. 2A are provided for all 115 traits (Additional file 2: Fig S3) and some examples are provided in Fig. 2B. The vast majority of PGS traits show good agreement between WGS and array-based PGS, even for traits with genetic architectures that include variants of large effect which can generate non-normal population distributions of PGS (e.g. Alzheimer’s Disease and Celiac Disease). To evaluate this quantitatively, regression of WGS PGS on PGS can be calculated across the 115 PGS traits. Figure 2C shows regression coefficients close to 1 for all genotyping instances (Additional file 1: Table S3). Another way to evaluate the technical reproducibility of PGS is based on percentile rank (Additional file 1: Table S4). For example, NA06997 was ranked in the 1 st percentile for height when considering the PGS calculated from WGS compared to the benchmark samples. The standard deviation of NA06997’s PGS percentile rank from the same chip is ~ 0.04, and the difference between maximum and minimum percentile rank is 0.33 of a percentile rank across all 64 replicates. Across 115 traits the mean range of NA06997’s PGS percentile is < 2 including across chips. The technical replicability is particularly strong between genotyping conducted on the same platform and processed through the same QC pipeline. While WGS PGS can be considered “ground-truth” because 30X WGS genotypes of each SNP should be accurately called, in fact 422 (0.0057%) and 443 (0.0060%) SNPs for NA06997 and NA07029 are missing from the list of 7.4 M SNPs used for calculation of PGS (Additional file 1: Table S5). Of these, 324 missing SNPs are shared between NA06997 and NA07029, which implies that the missingness reflects the reference genomes to which the WGS reads were mapped (a version of HRC). Hence, the array-based PGS could be more accurate depending on the contribution of the missing SNPs to the PGS (which will differ across traits). An investigation of the factors leading to the differences between PGS across genotyping instances are discussed below.

To provide additional evaluation of lcWGS, we compared PGS for 46 HSU samples genotyped with both lcWGS and GSA data, and both imputed to HRCr1.1. The mean correlation of the PGS across the two platforms among the 115 traits was 0.971 (sd = 0.062) (Additional file 2: Fig S4); only three traits were correlated lower than 0.9 (with mean = 0.973, and sd = 0.057). They are C4: 0.367 Celiac Disease 0.793 Primary biliary cirrhosis: 0.898 each of which has SNPs of large effect in the major histocompatibility complex (MHC) region. The MHC region is known to have a complex correlation structure and poor imputation of the SNPs in this region also explains the deviance of the lcWGS PGS for celiac disease and ulcerative colitis for the NA06997 (Fig. 2B). The correlation between the PGS when excluding the MHC region are 0.997, 0.997, and 0.994 for C4, Celiac Disease, Primary biliary cirrhosis respectively.

### Imputation

**Fig. 3:**
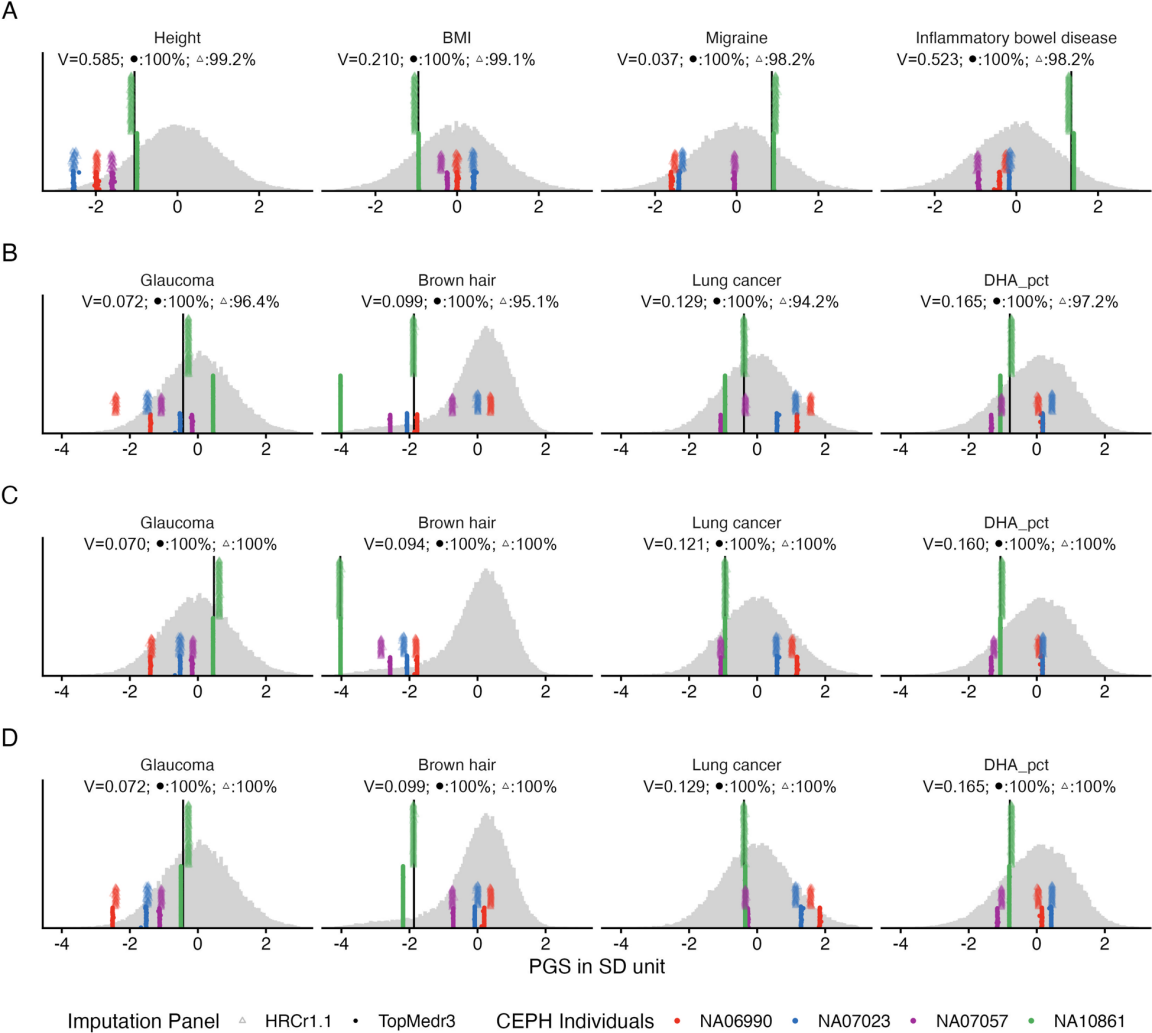
Imputation: PGS for four CEPH individuals genotyped on GSAv1 and imputed to two different reference databases. Underneath the trait headers, V is the sum of SNP variances; the percentages are the $PGS:QS$ quality scores (averaged over replicates) for the two imputation panels. **A** for most traits (see Additional file 2: Fig S5 and illustrated here by 4 example traits) the technical reproducibility (including across imputation reference) is very high; on average > 98% of the variance associated with the SNPs is captured. **B** the worst examples of technical reproducibility, where imputation reference panel has strong impact on the PGS, quantified by the PG:QS < 98%. **C** for the four traits in panel B, PGS are very similar across all replicates (as expected from part A) if we use only the overlap SNPs between the two imputed data (including in the PGS calculated from WGS; black line availale for only one individual), implying that missing SNPs rather than allele calling differences drives the differences shown in panel B. Since the SNP weights were derived from GWAS and LD matrices that used HRC imputation, the missing SNPs here are always in the TOPMed imputation, as indicated by the PGS matching the TOPMed imputation PGS in part B. While we have made the PGS similar here, the PGS are less accurate because critical SNPs have been excluded as indicated by the lower V compared to panel B. **D** The SNP weights are re-estimated using PGS-impute transferring weight from the missing SNPs to existent SNPs; the PGS estimated from TOPMed are now more similar to the PGS from HRC imputed SNPs and the V is recovered. The grey distribution of standardised PGS scores are the benchmark samples of European inferred ancestry from the Lifelines cohort imputed to HRC. Colours represent 4 CEPH individuals. Circles represent imputation to HRC release 1.1 reference panel and triangles represent imputation to the TOPMed reference panel. NB. As we show in part D, differences between the imputation panels owing to SNP missingness can be overcome. Both imputation panels generate high quality genotypes, and these results should not be used to infer preference of an imputation panel. BMI: body mass index: DHA_pct: Docosahexaenoic acid %. As a result of this research, the SBayesRC SNP weights now provided on the SBayesRC website for the 115 traits used here have been derived for the set of SNPs available in both HRC and TOPMed reference panels https://gctbhub.cloud.edu.au/software/gctb/#Download

Four CEPH samples (NA06990, NA07023, NA07057, NA10861) genotyped multiple times on the same array (Illumina GSAMD-24v1-0_20011747) by QIMRB were imputed to both HRCr1.1 and TOPMed reference panels. Of the 7.4 M SNPs with SBayesRC ${\widehat{\beta }}_{i}$ only 53 were missing from QIMRB HRCr1.1 imputed data (52 of which were also missing from HSU imputation). This high degree of overlap with the SBayesRC SNP list reflects that the SBayesRC LD reference was calculated from UK Biobank data which had been imputed to HRC data. In contrast, for TOPMed imputed data 211,126 (2.9%) and 15,738 (1.36%) of SBayesRC SNPs were missing from 7.4 M and 1.2 M sets, respectively. Consistent with Fig. 2 results, for the majority of traits (Additional file 2: Fig S5) the PGS for the same individual showed very high consistency across technical replicates when imputed to the same reference panel (illustrated for 4 traits in Fig. 3A). However, for a small number of traits the imputation panel had a strong impact on PGS results (see Fig. 3B for the most extreme examples).

Across the 115 traits, the median difference (across technical replicates) of standardized PGS between HRC imputed and TOPMed imputed data (for an individual) is 0.16 SD units (Additional file 2: Fig S6A). Considering the four traits with the largest differences in Fig. 3B raises the question if these differences arise either through missing SNPs or through differences in genotypes. To separate these sources of error we reduce the PGS calculation to a common SNP set present in all genotyping events including WGS (N = 7,124,899 SNPs; Additional file 1: Table S5); hence the number of SNPs in the TOPMed PGS is hardly changed, but the number of SNPs in the HRC PGS is reduced (by 2.9% for the 7.4 M set). We show that the PGS from the HRC imputation pipeline are now similar to the PGS from the TOPMed imputation (Fig. 3C) and the mean difference between the two PGS is now only 0.08 SD units (Additional file 2: Fig S6B). These results demonstrate that most of the differences shown in Fig. 3B reflect missing SNPs rather than genotyping errors. Supporting this conclusion, we note that in each case the PGS derived from WGS data has now moved reflecting that some SNPs contributing variance have been excluded in this demonstration as quantified by the reduced ${\widehat{V}}_{\cdot,\ j}$ in Fig. 3C compared to Fig. 3B. Hence, although in this exercise we achieved consistency of PGS across technical replicates there is loss of accuracy of the PGS because key SNPs are now missing from the PGS from both the pipelines. Next, we demonstrate that SNP weights for the SNPs missing in the TOPMed imputation can be redistributed to the SNPs available for an individual through the PGS-Impute algorithm (Fig. 3D). Through PGS-Impute the variance captured by SNPs for the TOPMed PGS is the same as for the standard SBayesRC SNP list, and consistency across the technical replicates is now mostly achieved. Any differences between technical replicates now reflect differences in genotype calls. For example, the TOPMed imputation PGS agrees with the WGS PGS for glaucoma while the HRC imputation agrees with the WGS PGS for brown hair colour (Fig. 3D). In both cases the differences in PGS can be explained by genotype differences at a small number of SNPs (13 for glaucoma and 11 for brown hair colour out of the top 1000 based on SNP effect size; Additional file 1: Table S6).

### Reporting

**Fig. 4:**
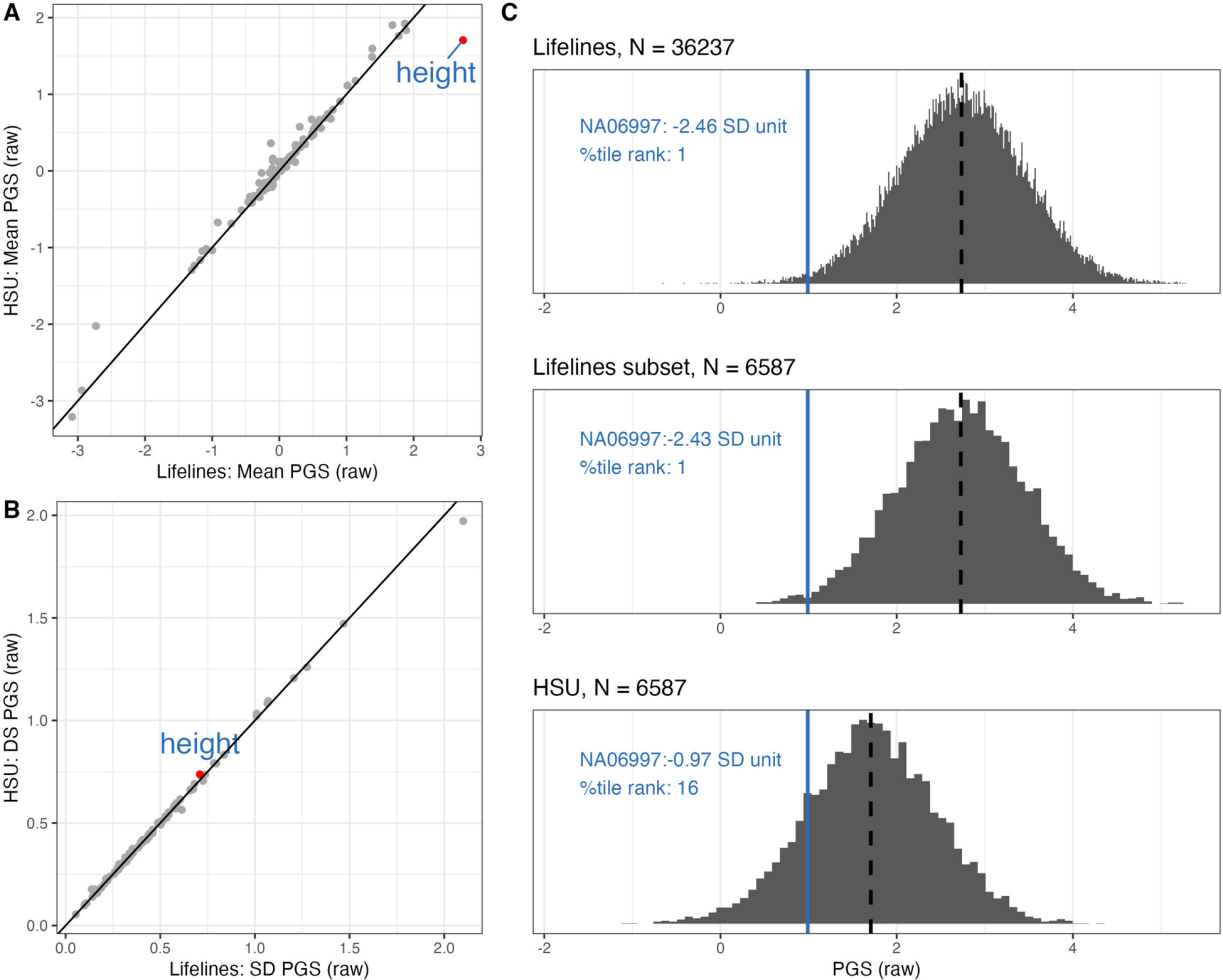
Reporting: Impact of benchmark cohort.** A** Mean **B** SD of PGS in raw SBayesRC units across 115 traits (each represented by a grey dot), x-axis: Lifelines healthy volunteer cohort (*N* = 36,237), y-axis: HSU cohort (*N* = 6,587) **C** For height, (the outlier in trait in A), the PGS of individual NA06997 is the same in SBayesRC units in all 3 panels (blue line), the benchmark mean (2.74 raw units) and SD (0.71) are the same for the full Lifelines cohort (top panel) and random subset (middle panel), but the mean is much lower (1.71) for the HSU cohort (bottom panel). Hence individual NA06697 is ranked in the 1 st percentile when benchmarked against Lifelines and 16th centile when benchmarked against HSU cohort

In real-world applications PGS values need to be presented in a way that is interpretable by health professionals and/or consumers. For example, individuals could be flagged as high genetic risk. The PGS therefore need to be benchmarked to the distribution of a representative population. The CEPH samples used in the study were of European ancestry. Here, the primary benchmark cohort is a sample of *N* = 36,237 unrelated individuals of inferred European ancestry from the Lifelines cohort from the northern provinces of the Netherlands. The PGS of each genotyping instance is scaled relative to the PGS distribution of this cohort, reporting the PGS either in standard deviation units or percentile rank. To demonstrate the impact of the benchmarking cohort we contrast the Lifelines cohort *N* = 36,237, with a random sample of *N* = 6,587 drawn from the Lifelines cohort, and with an independent inferred European ancestry sample of *N* = 6,587 from Australia (HSU cohort). We compared the mean and SD of the PGS from the Lifelines and HSU cohorts across the 115 traits (Fig. 4). All traits have consistent SD (Fig. 4B), but some traits showed different mean values (Fig. 4A). The biggest outlier was for height consistent with a previous report of people from Netherlands being genetically taller [33]; the mean PGS for the Lifelines cohort was 2.74 (s.e. 0.71) raw units, compared to 1.71 (s.e. 0.74) for the HSU cohort. The WGS derived PGS of NA06997 is −2.46 SD unit (1st percentile) when scaled to the Lifelines cohort, while scales to −0.97 SD unit (16th percentile) when benchmarked to the HSU samples (Fig. 4C).

Using the PGS derived from WGS, the rank of NA06997, NA07029, NA10861 across 115 traits the mean change in rank was 4.2 percentiles between the Lifelines and HSU cohorts. In comparison the mean difference in rank between using the full Lifelines cohort of *N* = 36,237 to the subset of *N* = 6,587 is very small, at 0.34 of a percentile across the 3 samples and 115 traits. These results demonstrate the impact of choice of PGS benchmarking cohort on risk stratification and we note that choice of benchmarking will require particular consideration in multi-ancestry studies (Table 2).

**Table 2:** PGS analytic validity checklist

• Provide a list of SNPs, their alleles, and the weights associated with the alleles, with a naming system for the list that makes them retrievable
• Provide the estimated variance associated with each SNP; this is easily achievable when SNP weights are derived from Bayesian methods as SNP weights are joint effect sizes, and so the estimated variances are (practically) independent and the variances sum to the SNP-based heritability (approximately). For SNP effect estimates not estimated jointly other approximations should be derived and justified. This provides a useful benchmark of how much variation can be captured by the PGS for a specific trait generated from the SNP genotypes available for an individual
• Identify SNPs of large effect (that are estimated to explain > 0.02% of variance) and genotype these directly rather than imputing them (e.g., include them in the custom content of a genotyping array)
• Report *V*_*j*_ which represents the total proportion of population variance attributable to the PGS for trait *j*, which is a useful benchmark for contrasting the relative importance of PGS across traits
• Provide detailed documentation of the genotype data generation, QC pipeline and imputation reference sample
• Provide a statement that all the SNPs in the PGS SNP list are included in the imputation reference sample
• Provide a PGS:QS statistic for each PGS for each individual as a quantification of the impact of SNPs missing for the individual
• Flag any PGS for an individual where the PGS:QS is less than 98% (this threshold may need to be confirmed in other data sets)

## Discussion

We exploited an opportunistic experimental design to demonstrate and quantify analytic validity of PGS. Our goal was to provide empirical data to assure PGS test accreditors and regulators that PGS tests are expected to have high technical validity. To explore technical validity, we used a fixed list of SNP weights for each trait, and hence ignore all discussion about the validity, utility or variability of PGS [4–6, 19], which are the most crucial criteria for clinical implementation. To explore technical validity, we first considered the genotyping and imputation steps of PGS variability (Fig. 1). We demonstrate very high reproducibility of PGS especially when generated from the same genotyping array, processed through the same QC and imputation pipeline. This high reproducibility was achieved from a research lab and hence should be further surpassed by commercial labs. We contrasted imputation to TOPMed and HRCr1.1 and showed little impact of imputation reference panel, consistent with other studies [34]. Of note, all imputation reference panels considered here were large (> 25 K) and since the HRCr1.1 is available for download to local servers, large imputation reference panels should always be achievable. It is possible that a commercial laboratory offering PGS may not have access to computing infrastructure needed for imputation to such large reference samples and so a commercial provider of PGS may need to demonstrate the validity of using a smaller reference sample size. This should be possible because reference panel size has most impact on imputation accuracy on imputation of rare variants [35], which are usually not needed to generate PGS.

Given a list of SNPs and their PGS weights, errors in PGS either come from missing SNPs or incorrect genotypes. In our research-environment analysis we find that most SNP missingness results from SNPs missing in the imputation reference sample (or WGS mapping reference) but present in the PGS SNP list. In practice, a company applying for accreditation of PGS tests will have a dedicated pipeline in which the imputation reference will be known and the SNP list for their PGS will be matched to the imputation reference list, hence missingness of SNPs will be minimised. Nonetheless, as a safeguard for each individual genotyped and for each PGS, a quality score (PGS:QS) can be calculated as a quantification of the impact of missing SNPs. We recommend that PGS:QS < 0.98 should be flagged.

One motivation for taking PGS into health settings is that the same set of genotype data can be used to calculate PGS for multiple traits. While the same SNPs may be missing for all traits for a particular person, the consequences of the missing SNPs may only be important for some PGS traits. We demonstrate that for SBayesRC SNP weights, a PGS-impute algorithm (requiring the SBayesRC SNP weights, LD matrix (as provided in the SBayesRC software) and SNP list of all non-missing SNPs) can generate PGS that recover the lost information from missing SNPs. This algorithm is likely more useful in research settings (e.g., for application of published SBayesRC weights to new data sets where not all SNPs are available), because when PGS calculations move into clinical applications, genotypes will be generated in a PGS-accredited environment where it will be desirable to establish a pipeline in which SNP missingness is avoided. The PGS-impute algorithm can be applied to PGS weights derived from other methods, but this should be evaluated with real data. One outcome of this research is to provide updated SBayesRC SNP weights on the GCTB website (see URL) which use the set of SNPs common across HRC and TOPMed imputation, which will help avoid problems of SNP missingness in PGS calculations in research settings.

Here, we show no gain for PGS accuracy from WGS data compared cheaper genotyping, consistent with a report in the context of GWAS discovery [36]. However, in research settings, there is now a trend away from genotyping arrays towards low coverage WGS. Justification for this trend is that lcWGS data are comparable in cost and efficiency for GWAS [37] and PGS analyses [9, 38] but also provide additional information, for example, estimation of mitochondrial count [39]. Moreover, Li et al. [9] concluded that lcWGS (at 1.2 × coverage) provides more accurate PGS (relative to WGS PGS) compared to genotyping array PGS. Their comparison was based on PGS for two traits, and they noted that their conclusion may change with larger imputation reference samples (such as HRC or TOPMed, as they used only the 1000 Genomes reference sample for imputation). Similarly, Homburger et al. [38] studied only 3 PGS traits, and used down-sampled WGS to investigate lcWGS PGS. Neither study included PGS traits with large effects from the MHC region. Our implementation of the latest GLIMPSE2 lcWGS imputation pipeline generates PGS with very good concordance with genotyping array PGS except for PGS that include SNPs with large effect size in the MHC region, a region of the genome that is particularly complex (Additional file 2: Fig S4). Our opportunistic experimental design does not allow a full exploration of the issues but based on our observations we would recommend that PGS based on lcWGS should demonstrate reproducibility across repeated lcWGS events for accuracy of MHC SNPs. The new “blended genome exome” (BGE) design [40] may alleviate some of the issues we observed, because the sequencing library is prepared to provide 30–40 × for exonic regions, while providing only 2–3 × for non-exonic regions. Thus, retaining the cost-effective advantage of lcWGS while providing high quality genotypes for a structurally complex region most likely to have SNPs of very large effect size in some PGS.

For a specific trait there is no technical variability in PGS attributed to the scoring step (Fig. 1), because the SNP allele weights are part of the definition of the PGS. Of course, over time discovery GWAS may have increased power or there may be improved methods to optimise SNP weights may become available so that the SNP allele weights for a PGS may be updated, and hence the PGS of an individual may change over time even though their DNA is constant. A traceable nomenclature system of PGS is needed for reporting of PGS to be transparent. We generated PGS for 115 traits for each genotyping instance to demonstrate empirically that the same genotype data can generate accurate PGS (compared to the gold standard of WGS) for some traits, but not others; the latter affected by missingness or inaccurate genotyping of particular SNPs. Last, we used different background reference panels to demonstrate that these can have impact on reporting of PGS, and hence the reference panel should also be traceable.

Our study only considered European ancestry samples. Improving PGS transferability is an important and active area of research [41] and is important for implementation of PGS in practice [42]. It is well documented that the TOPMed imputation reference provides more accurate imputation for people of non-European ancestry, given its larger sample size and ancestral representation [24]. Both TOPMed and HRC have a QC filter only retaining SNPs with a minor allele count of 5. However, PGS are based on common SNPs (e.g., > 1% in SBayesRC) which are ancient and hence common causal SNPs are likely to be shared across ancestries, and there is increasing empirical data to support this parsimonious expectation [43]. However, PGS derived from European ancestry GWAS are found to transfer increasingly poorly with genetic distance to other ancestries [44]. This is attributed to breakdown in the correlation between causal variants and variants with genotypes (i.e., differences in the local LD of genomic regions). Efforts to increase GWAS sample sizes in other ancestries, together with methodological approaches (e.g. using functional annotations) will help prioritise SNPs that are highly correlated to causal SNPs in all ancestries. The anticipated increase in sample sizes from diverse ancestries over time will support stronger evidenced-based conclusions.

## Conclusions

To summarise key take-home messages from our study, we provide a checklist for PGS analytic validity (Table 2). Our study was not specifically designed but uses opportunistically available data generated in our laboratories, mostly using data from only six European ancestry individuals genotyped multiple times. Hence, not all the important questions can be addressed, and not all questions were addressed equally (e.g., we had limited lcWGS data). Nonetheless, these empirical data provide novel and relevant results, which provide some insights for PGS providers, accreditors and regulators. The data presented are from research laboratories yet demonstrate high technical reproducibility of PGS. We have explored reasons for some examples of poor reproducibility of PGS from these research data and strongly believe that laboratories seeking accreditation for PGS will be able to establish pipelines which generate PGS with very high analytic validity.

## Supplementary Information

Additional file 1.

Additional file 2.

## Data Availability

WGS data for CEPH individuals: [https://www.internationalgenome.org/data-portal/data-collection/30x-grch38]. The genotype data for 46 samples lcWGS can be accessed through application here: [https://salsasgc.org/explore/]. Instructions for access to download the array genotype data for the CEPH controls generated by the University of Queensland HSU and by QIMR Berghofer can be found at https://doi.org/10.6084/m9.figshare.31797601. Haplotype Reference Consortium: Release 1.1 [https://ega-archive.org/datasets/EGAD00001002729]. Michigan imputation server: [https://imputationserver.sph.umich.edu/#]!. TOPMed imputation server: [https://imputation.biodatacatalyst.nhlbi.nih.gov/]. SBayesRC: [https://cnsgenomics.com/software/gctb/#Download]. SBayesRC(17) SNP weights for 115 traits used in this study and limited to the SNPs in common between HRC and TOPMed imputation reference panels can be downloaded from: [https://gctbhub.cloud.edu.au/data/SBayesRC/share/115traits/] or [https://gctbhub.cloud.edu.au/software/gctb/#Download]. All the coding scripts for this work are available in our Github website. [https://cnsgenomics.github.io/PGS_analytic_validity].

## Abbreviations

BMI: Body mass index
CEPH: Centre d'Etude du Polymorphism Humain (CEPH) samples (north-west European ancestry)
DHA_pct: Docosahexaenoic acid %
GCTB: Genome-wide Complex Trait Bayesian analysis, a software suite for Bayesian analysis used in individual level or summary statistic GWAS data
GWAS: Genome-wide association study
HREC: Human Research Ethics Committee
HSU: Human Studies Unit of the University of Queensland, Brisbane, Australia
lcWGS: Low coverage whole genome sequencing, refers to read depth of 0.5-4X (referring to the mean number of reads per base)
LD: Linkage disequilibrium
LDL: Low density lipoprotein cholesterol
MIA: Multivariate Index Assay
MSAC: Medical Services Advisory Committee (Australia)
NATA: National Association of Testing Authorities (Australia
NPAAC: National Pathology Accreditation Advisory Council (Australia) Multivariate Index Assays
PGS: Polygenic scores (generic for quantitative, binary or disease traits)
PRS: Polygenic risk scores, PGS for disease traits
PUFA: Polyunsaturated fatty acids
QC: Quality control
QIMRB: QIMR Berghofer Medical Research Institute, Brisbane, Australia
SBayesRC: Summary-statistics based Bayesian multiple linear regressions that calculates joint SNP effect sizes from GWAS summary statistics; the SBayesR approach models effect size distributions in an approach that allows optimisation for any underlying genetic architecture, SBayesRC further improves optimisation of SNP weights by using functional annotations of SNPs in the algorithm
SNP: Single nucleotide polymorphism
TGA: Therapeutic Goods Administration (Australia)
WGS: Whole genome sequencing (usually expecting read depth of 30X)
