# Supplementary material for: Empirical evaluation of analytic validity of polygenic scores
Source: Genome Med. 2026 Apr 23;18:81. doi: 10.1186/s13073-026-01654-6 (PMC13238075; doi:10.1186/s13073-026-01654-6)
Supplement: Supplementary file 1 — Additional file 1. [file 13073_2026_1654_MOESM1_ESM.pdf]

Empirical evaluation of analytic validity of polygenic scores

Lin et al

Supplementary Materials

Supplementary Note

UQ genotype data pipeline

For CEPH samples DNA was received as lymphoblastoid cell lines. For in-house studies (which provided the secondary benchmark data set) DNA was extracted in the UQ Human Studies Unit (HSU) following best practice guidelines from blood or saliva. The HSU attained formal international accreditation in 2023 for sample biobanking (Canadian Tissue Repository Network BRC-00588 number). Genotyping using Illumina iscan was conducted in-house in batches of 90 to 200 samples.

We used common best practice to QC the raw data (see Github code), including exclusion of SNPs with a minor allele frequency <0.01, SNP call rate 0.95, and Hardy-Weinberg equilibrium test ($p<1x10^{-6}$). All samples had a genotyping call rate above 0.95. Our in-house QC pipeline is customized to process batches with small sample size uses a list of 488,479 SNPs genotyped which we have previously shown to be consistently genotyped with high quality from the GSAv3 array (identified from analysis of 8,859 samples with array data generated in 75 batches). For each batch these SNPs were extracted from Illumina idat files, alleles flipped to the positive strand. The high-quality SNPs were retained for phasing and imputation from this chip. Similar pipelines were made for each version of the array (see Table 1 for numbers of high-quality SNPs used for imputation). Data were converted to vcf format with reference allele aligned to the imputation reference data using BCFtools(1). The data were phased using Eagle2(2), and imputed with Impute5(3). Both phasing and imputation used the Haplotype Reference Consortium (HRC)(4) Release 1.1 (HRCr1.1) imputation panel, which has 27,165 samples. For memory-efficient storage, genotypes were converted from dosage to their best-guess genotypes regardless of INFO score (where INFO score is the ratio of observed genotype variance to expected variance, which is the binomial variance calculated from allele frequencies, such that poorly imputed SNPs are expected to have lower observed compared to expected variance). Missing SNP IDs were set to physical location, and duplicate SNP IDs had a suffix added to their ID to identify them.

The Axiom SARS-COV2 (Ramaciotti) chip was used for one batch of 96 samples, which included both NA07029 and NA06997 samples. We followed common best practices for QC of the raw data. All samples had a genotyping call rate above 95%. A list of 500,112 high quality SNPs was used for phasing and imputation from this chip.

Whole genome sequencing pipeline

High coverage WGS data for the CEPH individuals were downloaded from the International Genome Sample Resource (https://www.internationalgenome.org/data-portal/sample). The data were already in aligned and mapping format (BAM) against build 38. Variants were called using GATK(5) with base-pair resolution of BP and dbSNP annotation. Missing SNP IDs were recovered by comparing to the dbSNP reference. The SBayesRC SNP sets used for PGS calculations were extracted using dbSNP ID.

QIMRB QC and imputation

The CEPH samples additional randomly selected genotyped samples were selected from each of the arrays contributed (IGSAv3, I610Qv1, I660W1, see **Supplementary Table 1**) to make batches of 200 samples. SNPs which were removed if they had SNPs with a minor allele

frequency <0.01, SNP call rate 0.975, and Hardy-Weinberg equilibrium test ($p<1x10^{-6}$). Data were submitted to both TOPmed server with TOPmed r3 imputation panel, and Michigan server with HRCr1.1 panel. Downloaded vcf files were converted to PLINK format. When no dbSNP ID was found, the SNPID was set to physical location. Duplicate SNP IDs are distinguished by adding a suffix, while keeping the SNP that have the same pair of alleles vs. the predictors intact.

Low pass sequencing

As a trial for the validity of lcWGS, 46 samples previously genotyped with Illumina GSAv2+MD were submitted for lcWGS including NA06997. Library preparation was conducted in house at the UQ Human Studies Unit and submitted for sequencing in UQ sequencing facility. The 46 samples were pooled with multiplexing, and sequenced in one lane, setting paired end 150bp reads on a SP flowcell using NovaSeq Illumina. Raw data were transferred to us in FastQ format. All samples passed FastQC quality threshold, and the reads were mapped to human genome assembly build 37 using the bwa in GATK. The GATK best practice pipeline was applied, (sort using sambamba, mark-dup using GATK, Base Quality Score Recalibration using GATK). Missing SNPs in data were imputed using GLIMPSE2(6) against the downloaded HRCr1.1 imputation panel.

GLIPMSE2 uses the reference data to decide the chunks required to speed up imputation in parallel jobs. It connects the imputed chunks into one data per sample in VCF format. The SNP list in imputed data is the same as in the HRC imputation panel.

SBayesRC PGS

To generate SBayesRC SNP weights a reference LD matrix is needed. The default European ancestry high density of LD matrix of 7,356,518 SNPs available in GCTB is calculated from UK Biobank individuals who have been imputed to the HRC reference which is in genome build 37.

GWAS summary statistics were converted into a standard format, back calculating variables if needed and using reference data allele frequency if missing.

**Column Name**	**Description**
SNP	SNP ID
A1	Effect allele
A2	Reference allele
freq	Frequency of effect allele A1
b	Effect size (beta coefficient)
se	Standard error of beta
p	P-value for association
N	Sample size

For GWAS summary statistics missing dbSNP IDs are matched to dbSNP IDs with the physical position and both alleles based on the build. We exclude SNPs with large difference of allele frequency comparing to the LD reference data. When GWAS summary statistics are provided without allele frequency (p), we assume the same allele frequency as in the LD reference data. When per SNP sample size is available, we exclude the SNPs with sample size that differ >3SD of mean sample size. For a small number of GWAS summary statistics data sets, the marginal effect size of SNPs and the standard error are not reported in the summary statistics, but z is reported. We use these formulas to compute b and se.

$$b = \frac{z}{\sqrt{2 \times \mathrm{p} \times (1 - \mathrm{p}) \times (N + z^2)}}$$

$$se = \frac{1}{\sqrt{2 \times \, \mathrm{p} \times (1 - \mathrm{p}) \times (N + z^2)}}$$

SBayesRC was applied to each GWAS data set using default settings for most traits which generates SBayesRC SNP weights for 7.4M SNPs. When the coverage of a GWAS data is less than 70% of 7.4M SNPs, we used only the 1M of HapMap3 SNP. SNP weights are effect sizes of SNPs when fitted jointly.

PGS were calculated in PLINK using the –score sum command.

<u>PGS-impute</u>

Let '1' denote the full set of SNPs in the PGS list and '2' denote the subset of SNPs available in the genotypes of prediction individuals. Given the effect estimates of all SNPs ($\widehat{\boldsymbol{\beta}}_{\mathbf{1}}$), the goal is to obtain $\widehat{\boldsymbol{\beta}}_{\mathbf{2}}$ that can give the same PGS:

$$\mathbf{X}_{\mathbf{2}}\widehat{\boldsymbol{\beta}}_{\mathbf{2}} = \mathbf{X}_{\mathbf{1}}\widehat{\boldsymbol{\beta}}_{\mathbf{1}}$$

where $\mathbf{X}$ is the genotype matrix coded as 0/1/2. Multiplying both sides by $\mathbf{X}_{\mathbf{2}}'$ gives

$$\mathbf{X}_{\mathbf{2}}'\mathbf{X}_{\mathbf{2}}\widehat{\boldsymbol{\beta}}_{\mathbf{2}} = \mathbf{X}_{\mathbf{2}}'\mathbf{X}_{\mathbf{1}}\widehat{\boldsymbol{\beta}}_{\mathbf{1}}$$

which is equivalent to

$$\mathbf{D}_{\mathbf{2}}\mathbf{R}_{22}\mathbf{D}_{\mathbf{2}}\widehat{\boldsymbol{\beta}}_{\mathbf{2}} = \mathbf{D}_{\mathbf{2}}\mathbf{R}_{21}\mathbf{D}_{\mathbf{1}}\widehat{\boldsymbol{\beta}}_{\mathbf{1}}$$

Where $\mathbf{R}$ is the LD correlation matrix and $\mathbf{D}$ is the diagonal matrix of the sqrt root of SNP heterozygosity ($D_{ii} = \sqrt{2p_i(1 - p_i)}$ with p_i being the minor allele frequency of SNP i). Hence, we can estimate $\widehat{\boldsymbol{\beta}}_{\mathbf{2}}$ by

$$\widehat{\boldsymbol{\beta}}_{\mathbf{2}} = \mathbf{D}_{\mathbf{2}}^{-\mathbf{1}}\mathbf{R}_{\mathbf{22}}^{-\mathbf{1}}\mathbf{R}_{21}\mathbf{D}_{\mathbf{1}}\widehat{\boldsymbol{\beta}}_{\mathbf{1}}$$

In practice, we compute $\widehat{\boldsymbol{\beta}}_{\mathbf{2}}$ for a window of 1,000 SNPs at a time and with a pseudo-inverse of $\mathbf{R}_{22}$ computed from eigenvectors that explain at least 99.5% of variance in LD.

We note that our PGS-impute method differs from ImpG (7). ImpG imputes marginal SNP effects for missing SNPs using a multivariate normal approximation to GWAS summary statistics, with their covariance structure determined by LD. In contrast, our approach operates on joint SNP effects estimated from SBayesRC, which are conditional on the SNPs included in the model. Our objective is not to infer causal effects for missing SNPs, but to re-map joint effects from the available SNP set in a manner that preserves the polygenic score. Specifically, we use LD information to project the joint effects estimated from the full SNP set onto the subset of SNPs present in the prediction samples, such that the resulting PGS remains consistent before and after SNP effect mapping. This mapping is performed within windows of 1,000 consecutive SNPs, reflecting the local decay of LD.

Lifelines cohort

Lifelines is a multi-disciplinary prospective population-based cohort study examining in a unique three-generation design the health and health-related behaviours of 167,729 persons living in the North of the Netherlands. It employs a broad range of investigative procedures in assessing the biomedical, socio-demographic, behavioural, physical and psychological factors which contribute to the health and disease of the general population, with a special focus on multi-morbidity and complex genetics. Here, we use genotyping from the first genotyping batches selected unrelated individuals.

Figure S1. Criteria for selection of traits for PGS in this study.
Labelled traits were excluded, based on SNP-based heritability < 0.05 and SBayesRC re-run correlation < 0.85 (which indicates instability in the SNP weights). 115 traits remain. When SNP-based heritability is high, but re-run correlation is low then it usually implies that some SNP effect weights are very large. In real implementation of PGS for such traits the SNPs of large effect size would always be directly genotyped.

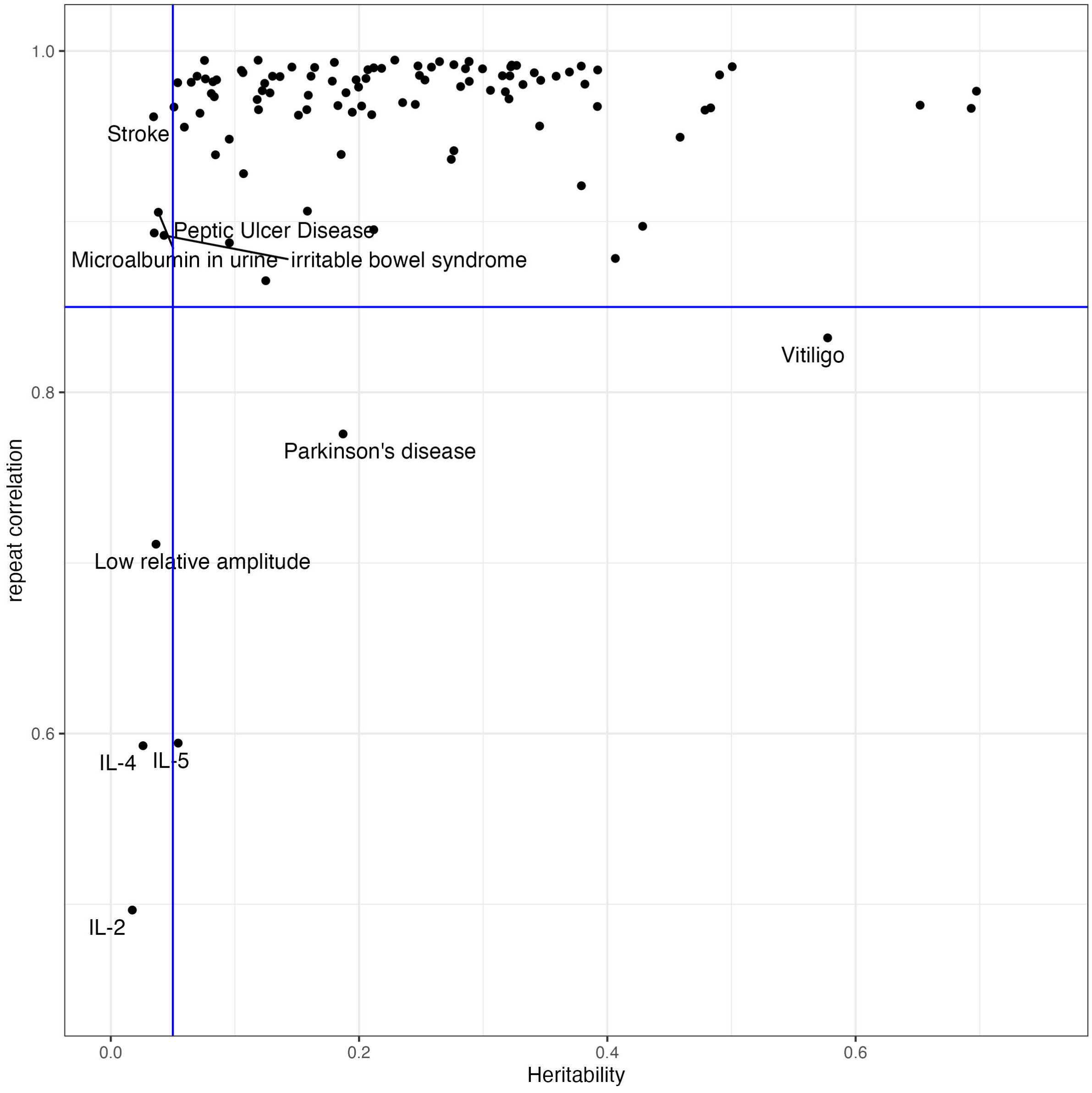

Figure S2. Relationship between SBayesRC SNP-based heritability and $\widehat{V_{\cdot,j}}$,sum of variances attributed to each SNP across all SNPs, for each trait (j)

SNP-based heritability is on the observed scale i.e., has not been converted to the liability scale.

Each dot is the value for a trait

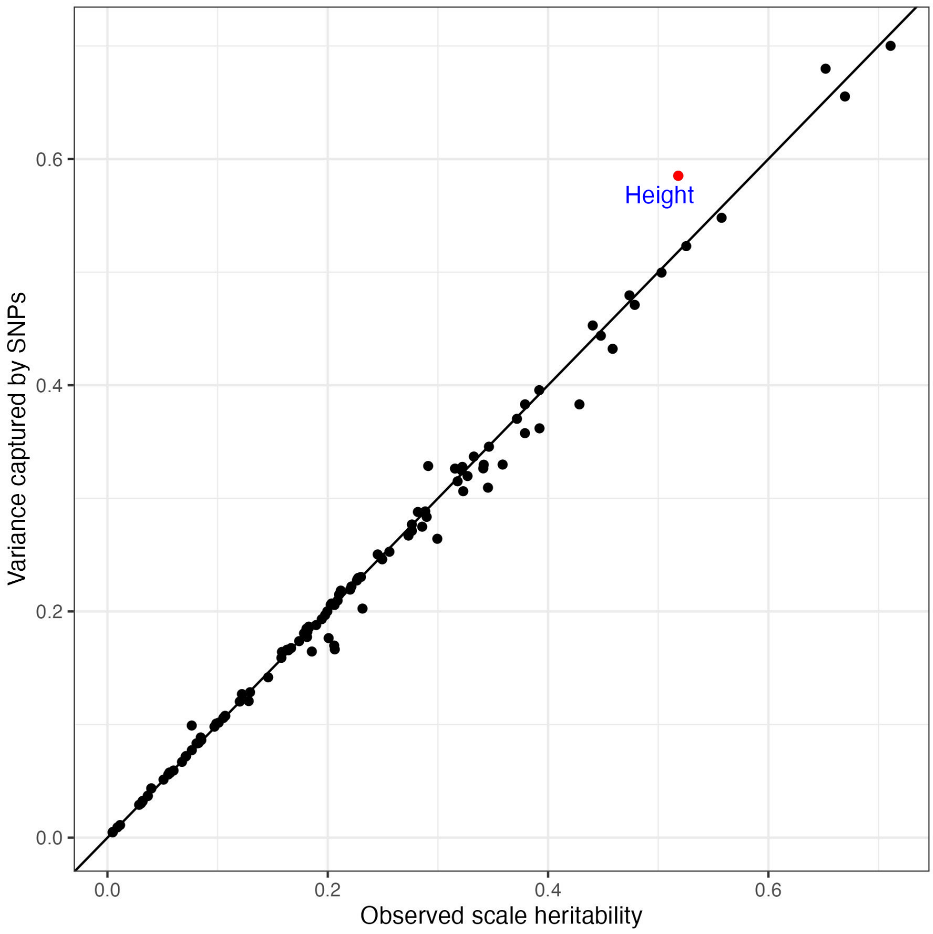

Figure S3. As for Figure 2A and 2B are provided for all 115 traits.

PGS for for CEPH individuals NA07029 (circles) and NA069977 (triangles) genotyped on different platforms (colours). See Supplementary Table 2 for a description of the traits, and definition of acronyms. The grey distributions of standardised PGS scores are from 36,237 benchmark samples of European inferred ancestry from the Lifelines cohort. The black vertical lines are PGS calculated from 30x WGS.

A. 32 Binary traits collected from different studies.

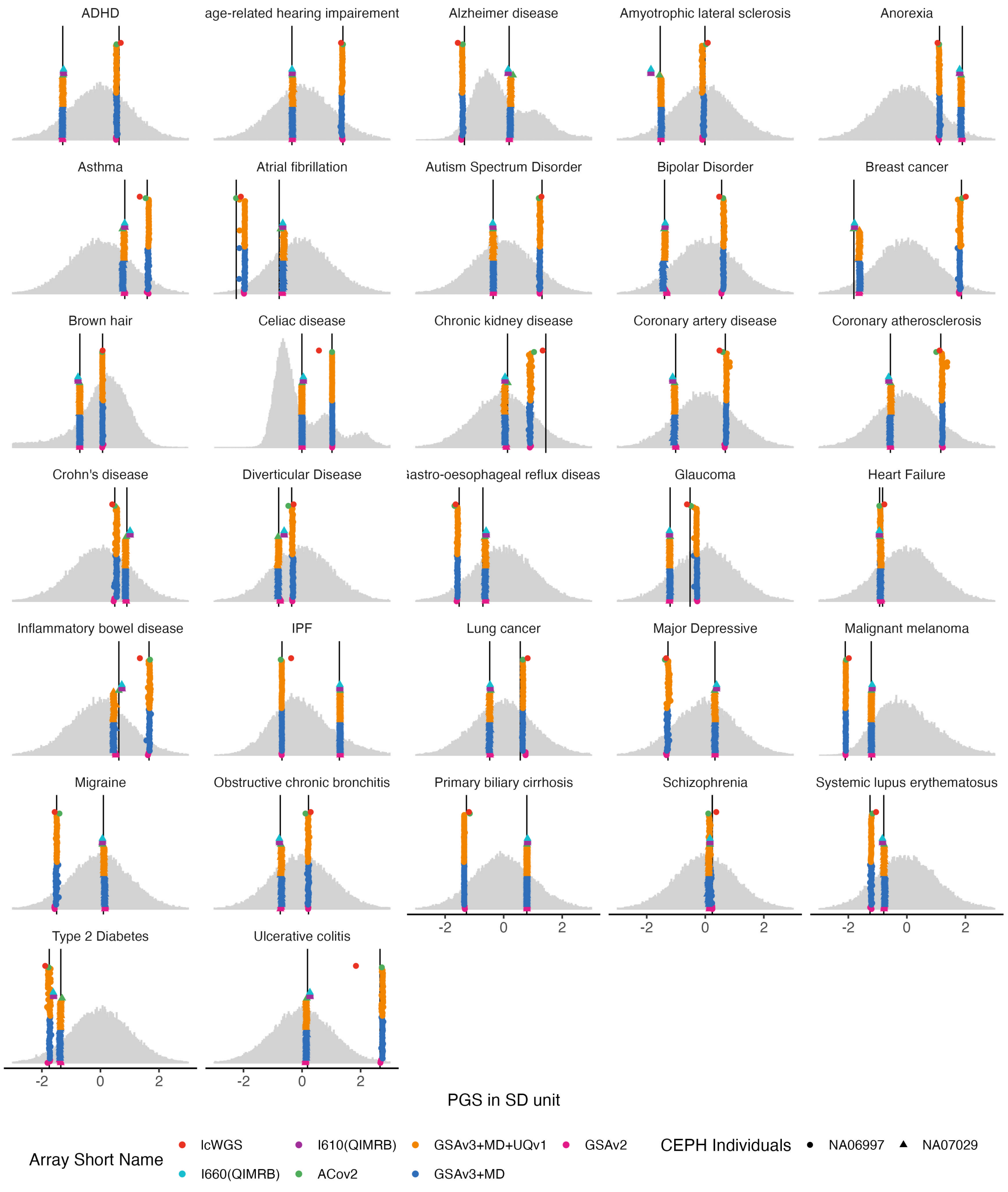

B. 23 quantitative traits collected from different studies.

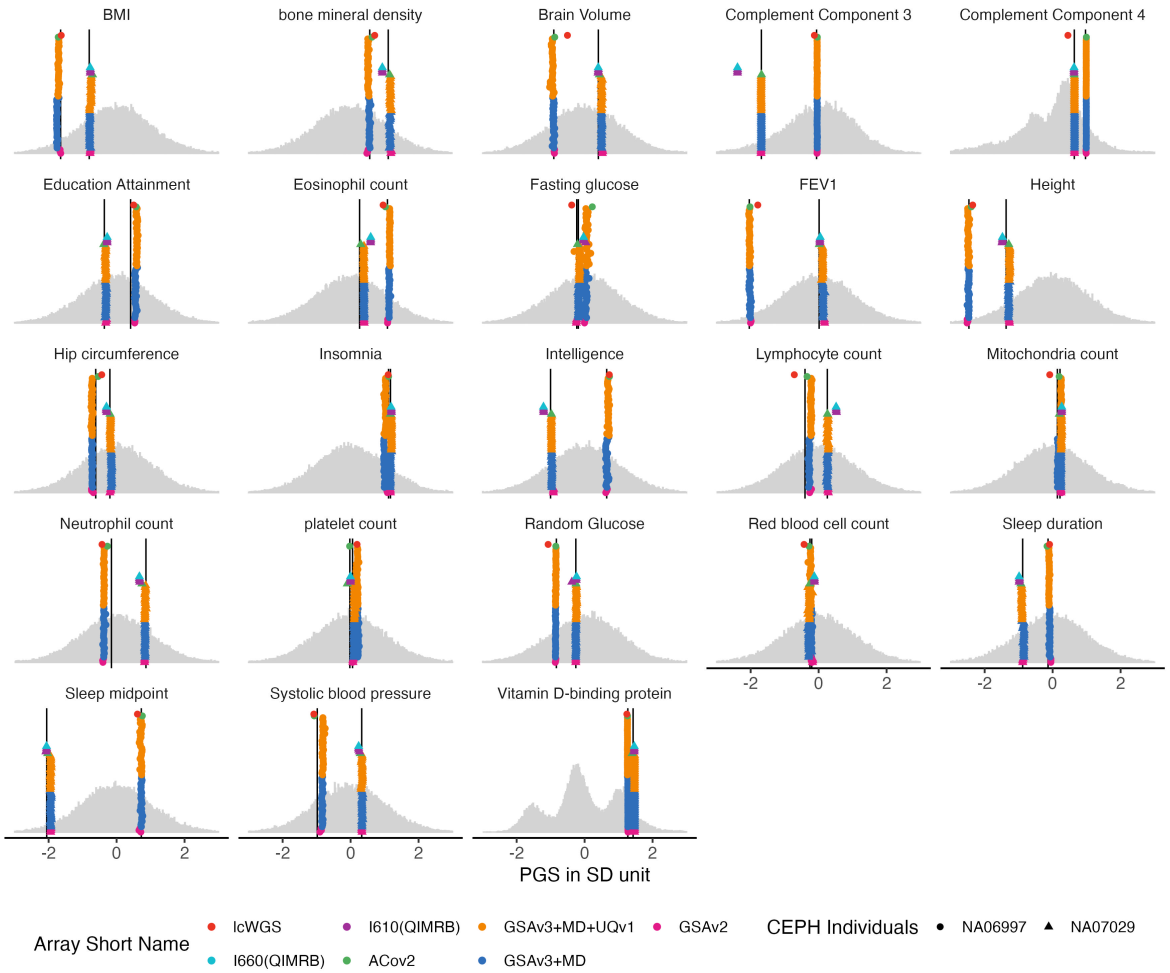

C. 33 traits selected from the 35 urine and blood biomarker in UK Biobank study Sinnott-Armstrong et al., 2021.

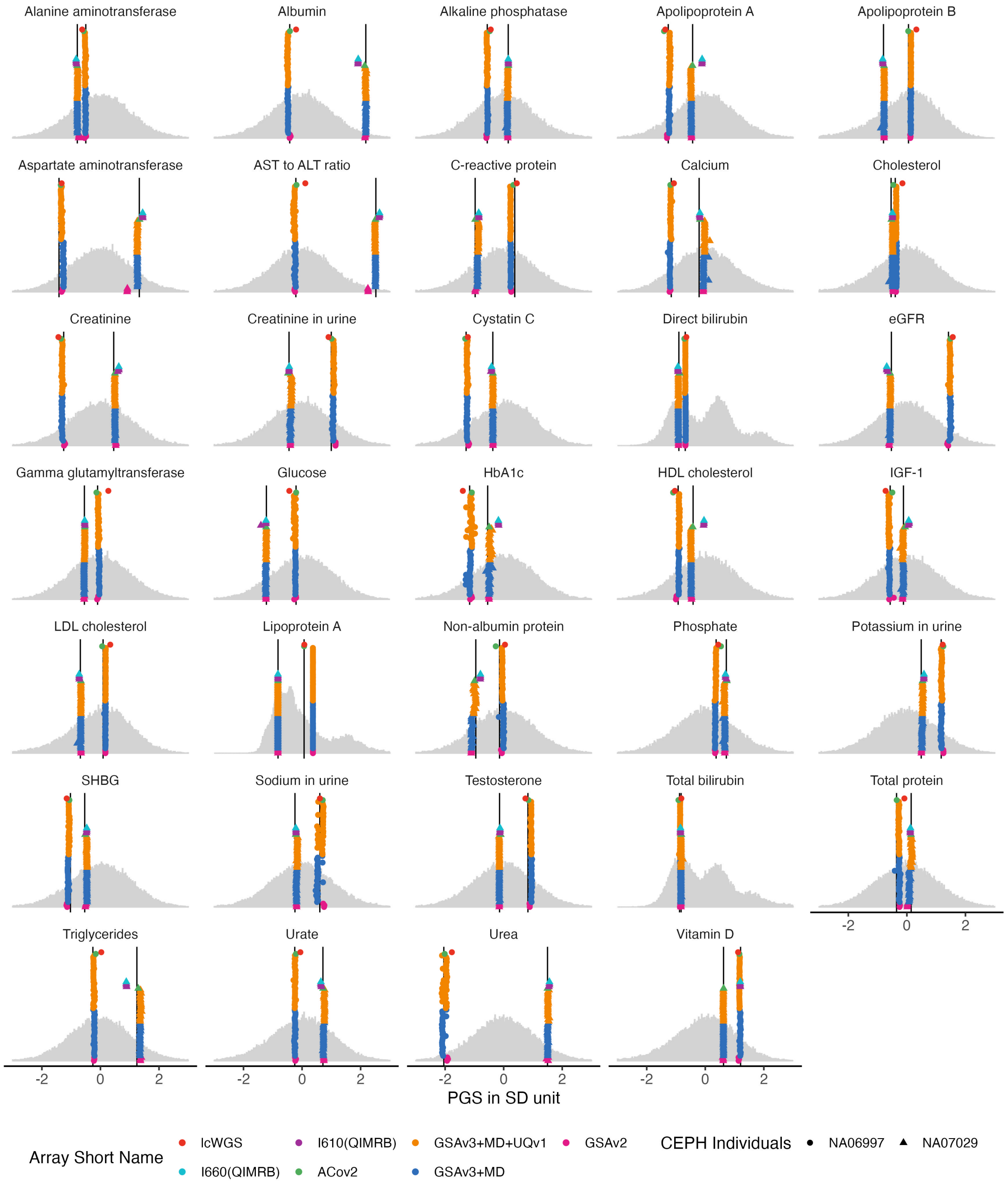

D. 16 Fatty Acid traits from the study Borges et al., 2022.

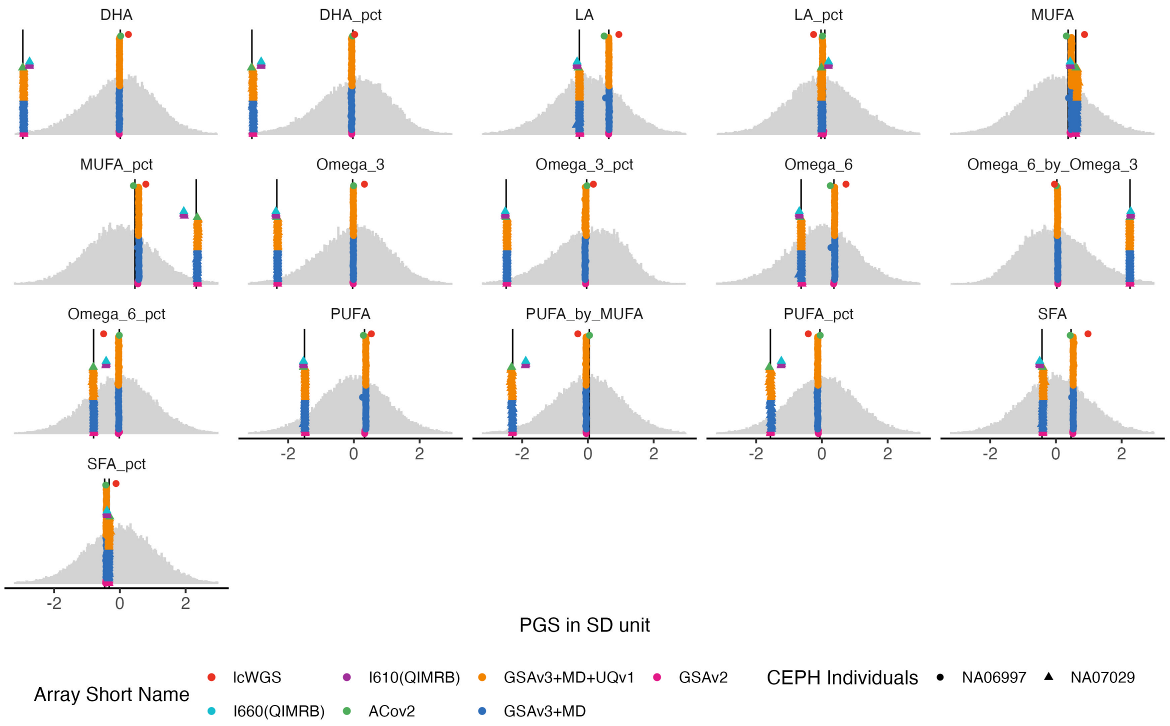

E. 10 Plasma Proteomics related traits from the study Sun et al., 2023

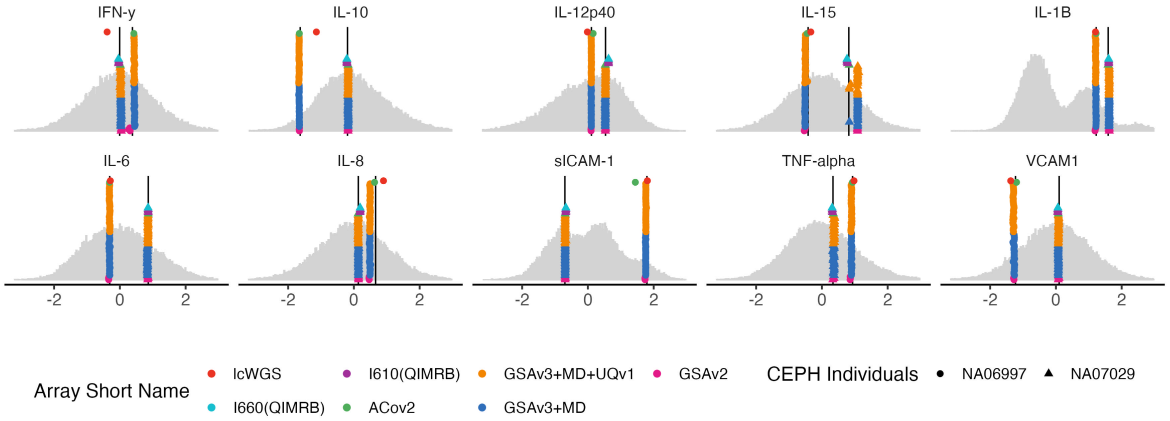

Figure S4, PGS profiled for 46 individuals using data from lcWGS vs from GSA chip.
Line in each plot is y=x, cor is the correlation. Each dot represents the PGS for an individual estimated from genotype data from the two technologies.
A. 32 Binary traits collected from different studies.

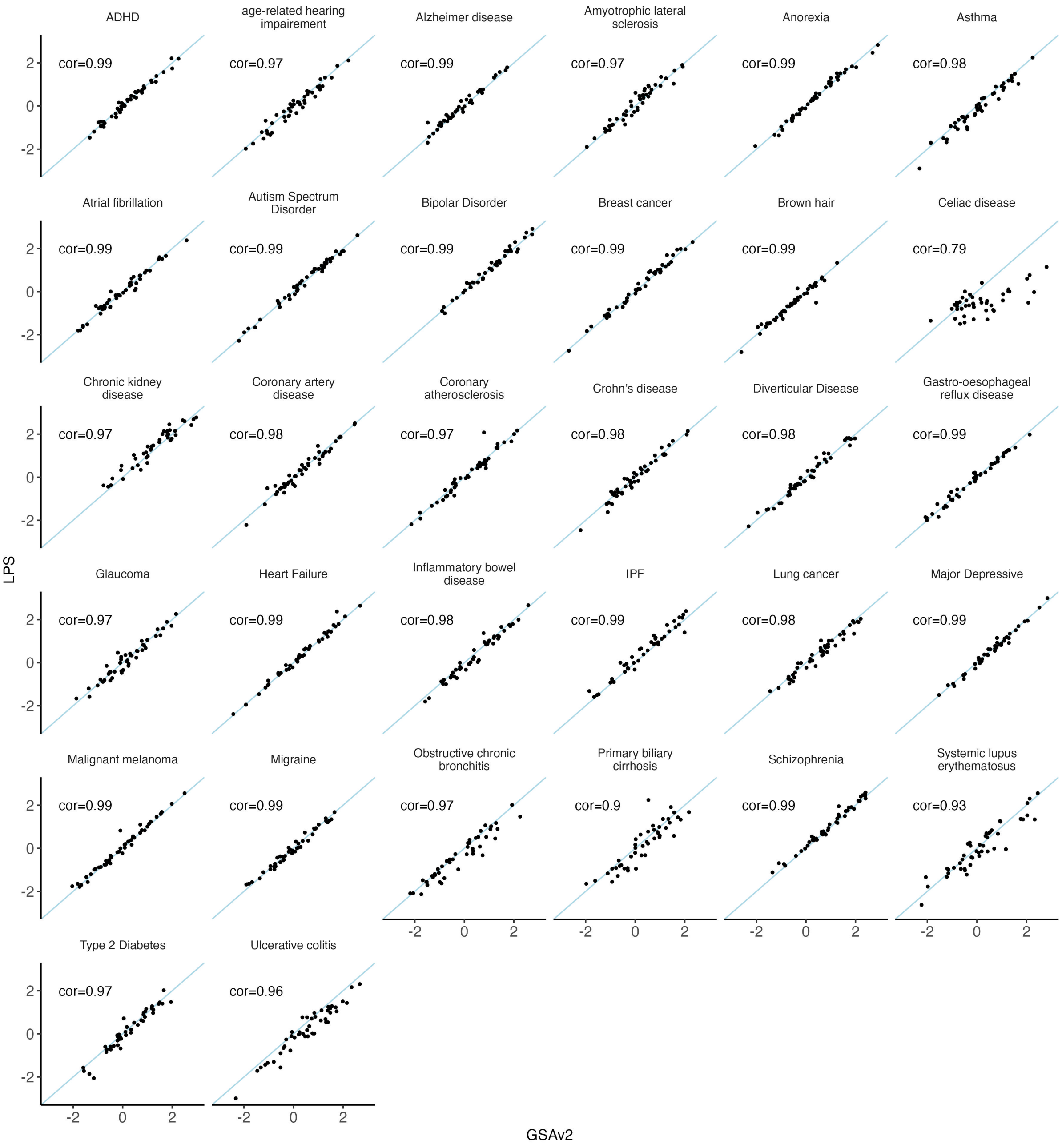

B. 23 quantitative traits collected from different studies.

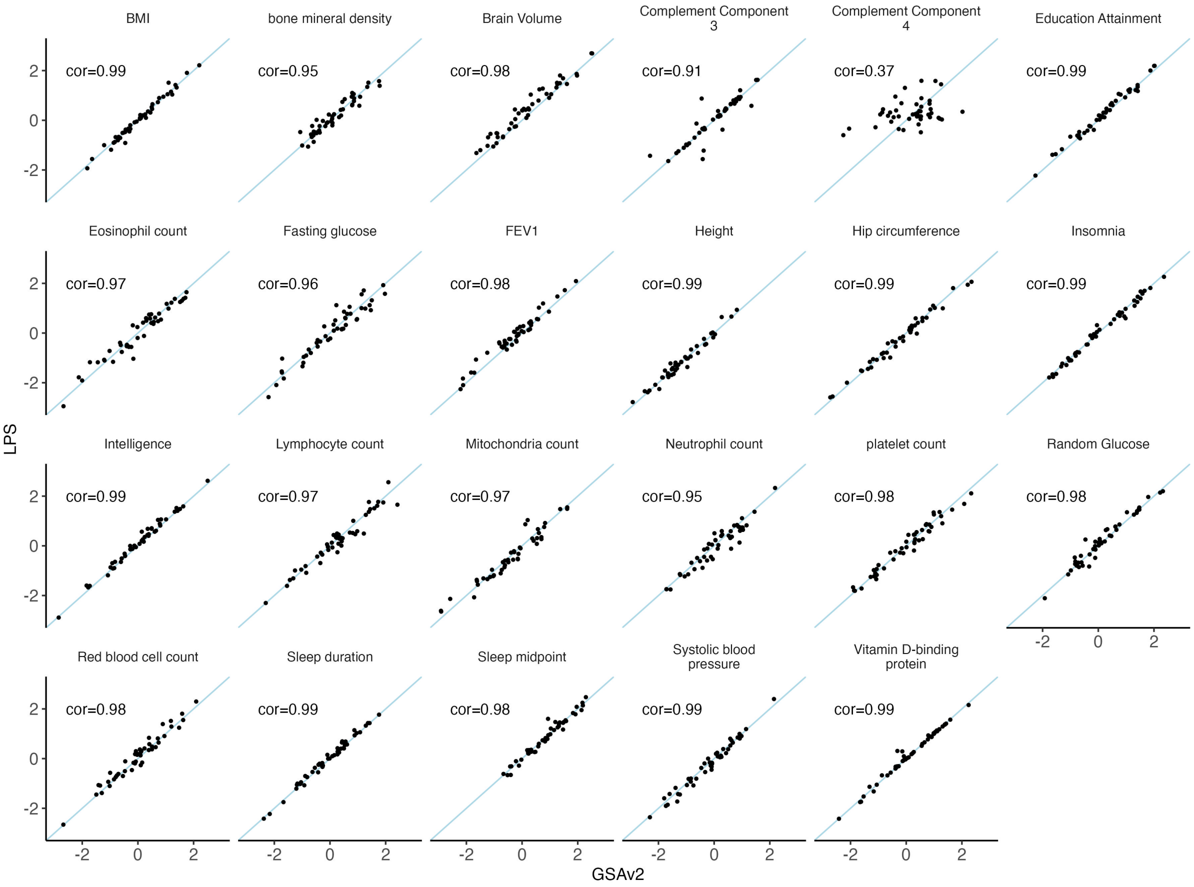

C. 33 traits selected from the 35 urine and blood biomarker in UK Biobank study Sinnott-Armstrong et al., 2021.

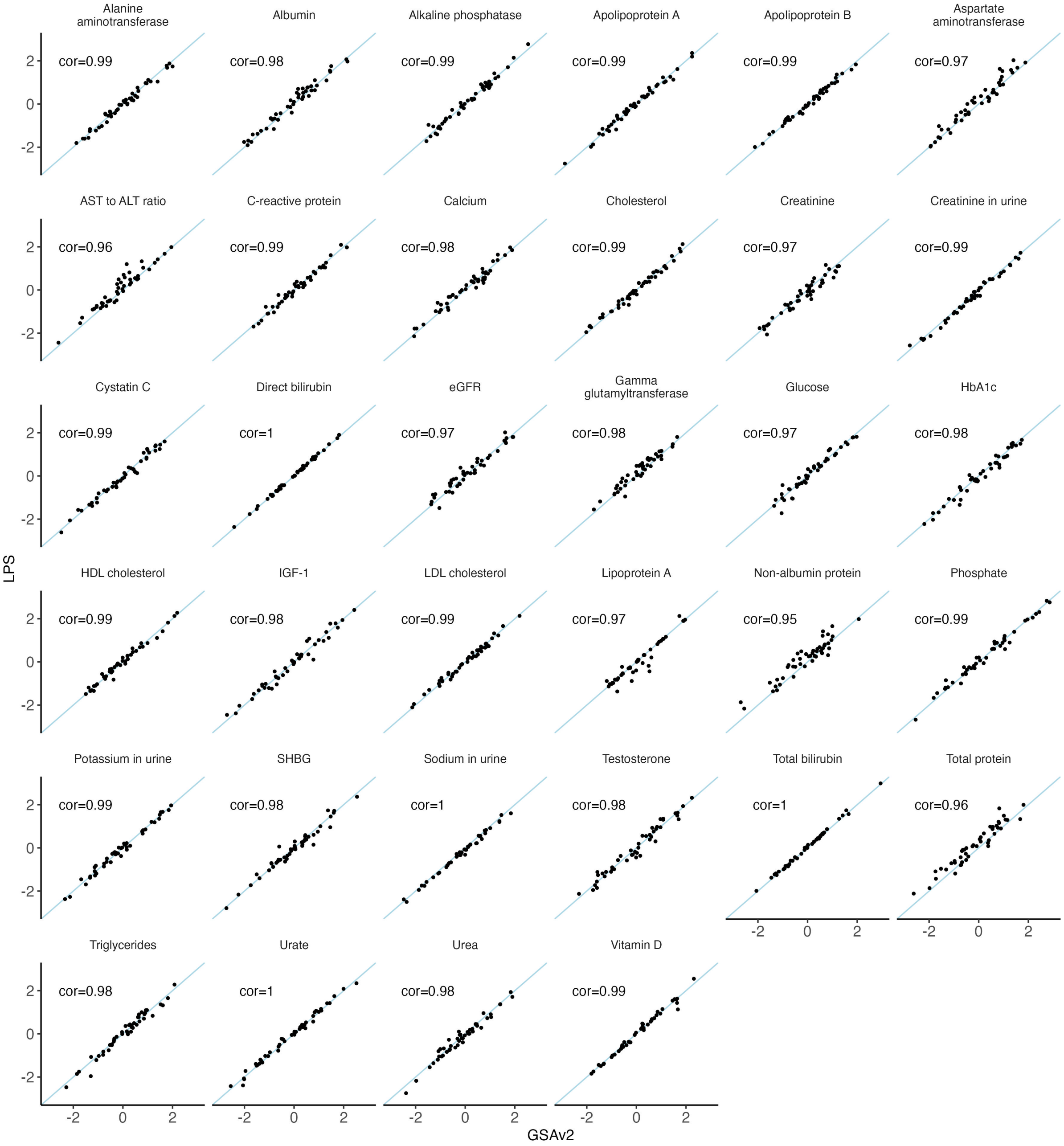

D. 16 Fatty Acid traits from the study Borges et al., 2022.

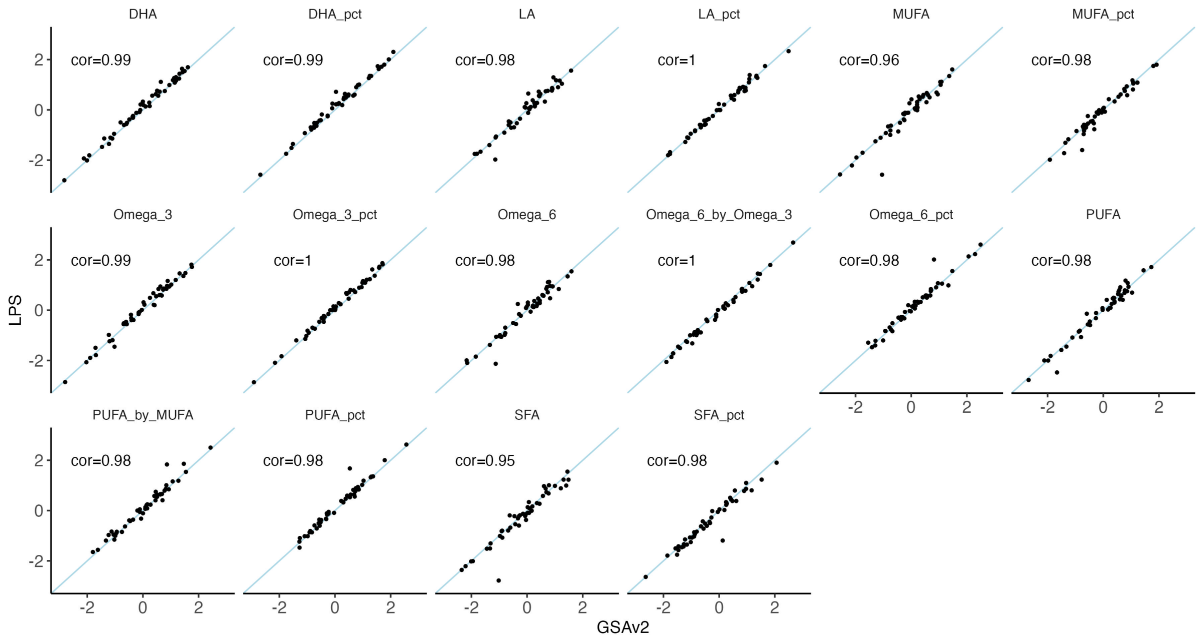

E. 10 Plasma Proteomics related traits from the study Sun et al., 2023

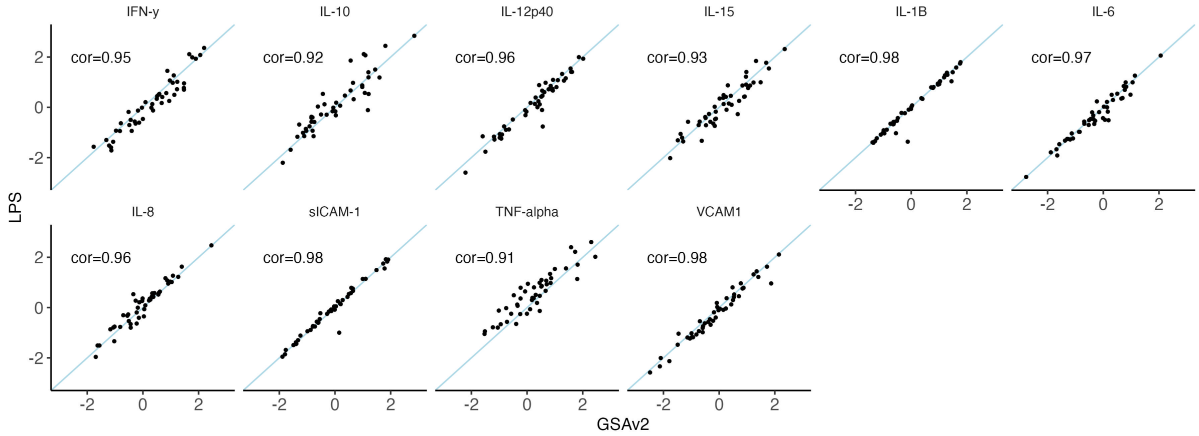

F. PGS estimated for each of 115 traits from lcWGS imputed genotyped vs GSAv3 imputed genotypes, with one plot for each of 46 people.

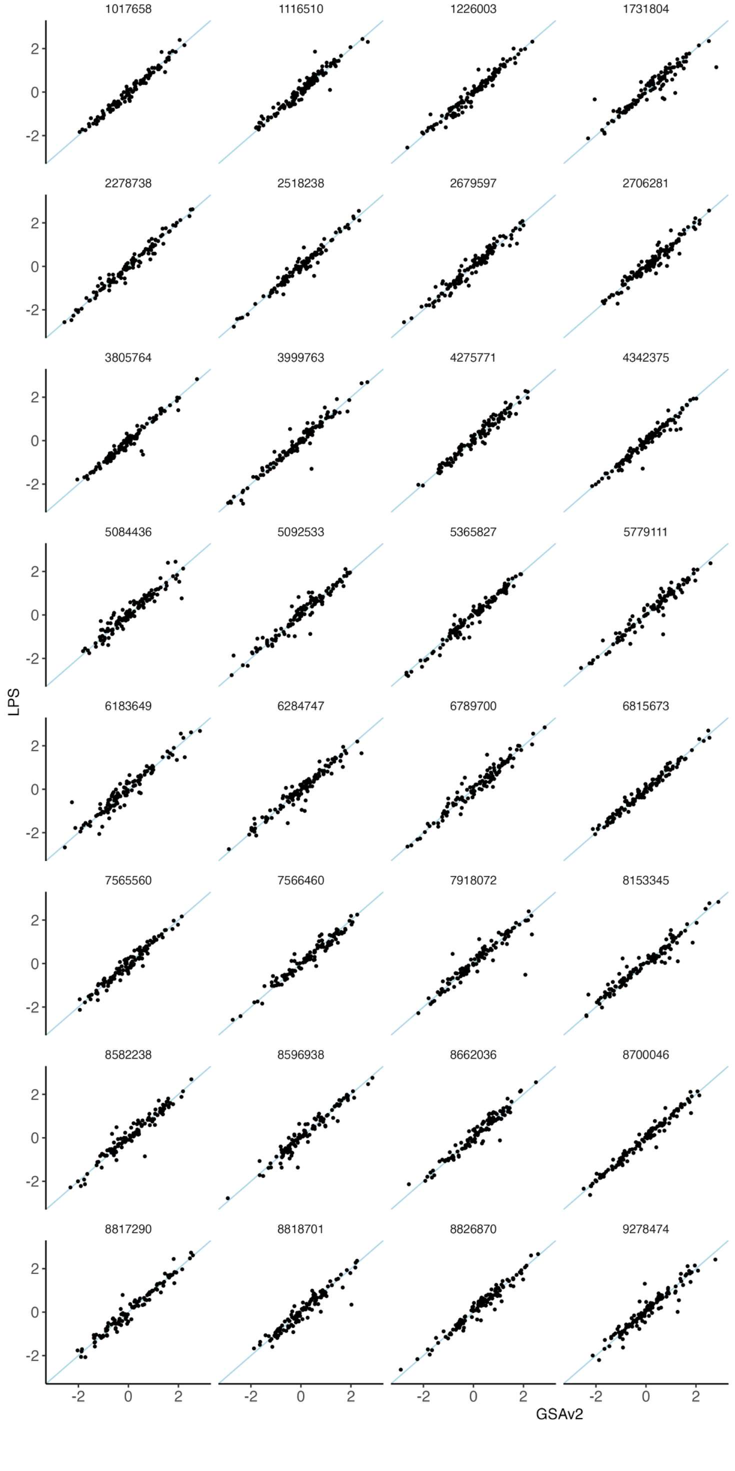

Figure S5. As for Figure 3A and 3B provided for all 115 traits.
The grey distribution of standardised PGS scores are the benchmark samples of European inferred ancestry from the Lifelines cohort imputed to HRC. Colours represent 4 CEPH individuals. Circles represent imputation to HRC release 1.1 reference panel and triangles represent imputation to the TOPMed reference panel. NB. As we show in Figure 3 D, differences between the imputation panels owing to SNP missingness can be overcome. Both imputation panels generate high quality genotypes, and these results should not be used to infer preference of an imputation panel. There is more missingness in the TOPMed imputation because SBayesRC results were generated from data stes using HRC imputation. As a result of this research, the SBayesRC SNP weights now provided on the SBayesRC website for the 115 traits used here have been derived for the set of SNPs available in both HRC and TOPMed reference panels https://gctbhub.cloud.edu.au/software/gctb/#Download

A. 32 Binary traits collected from different studies.

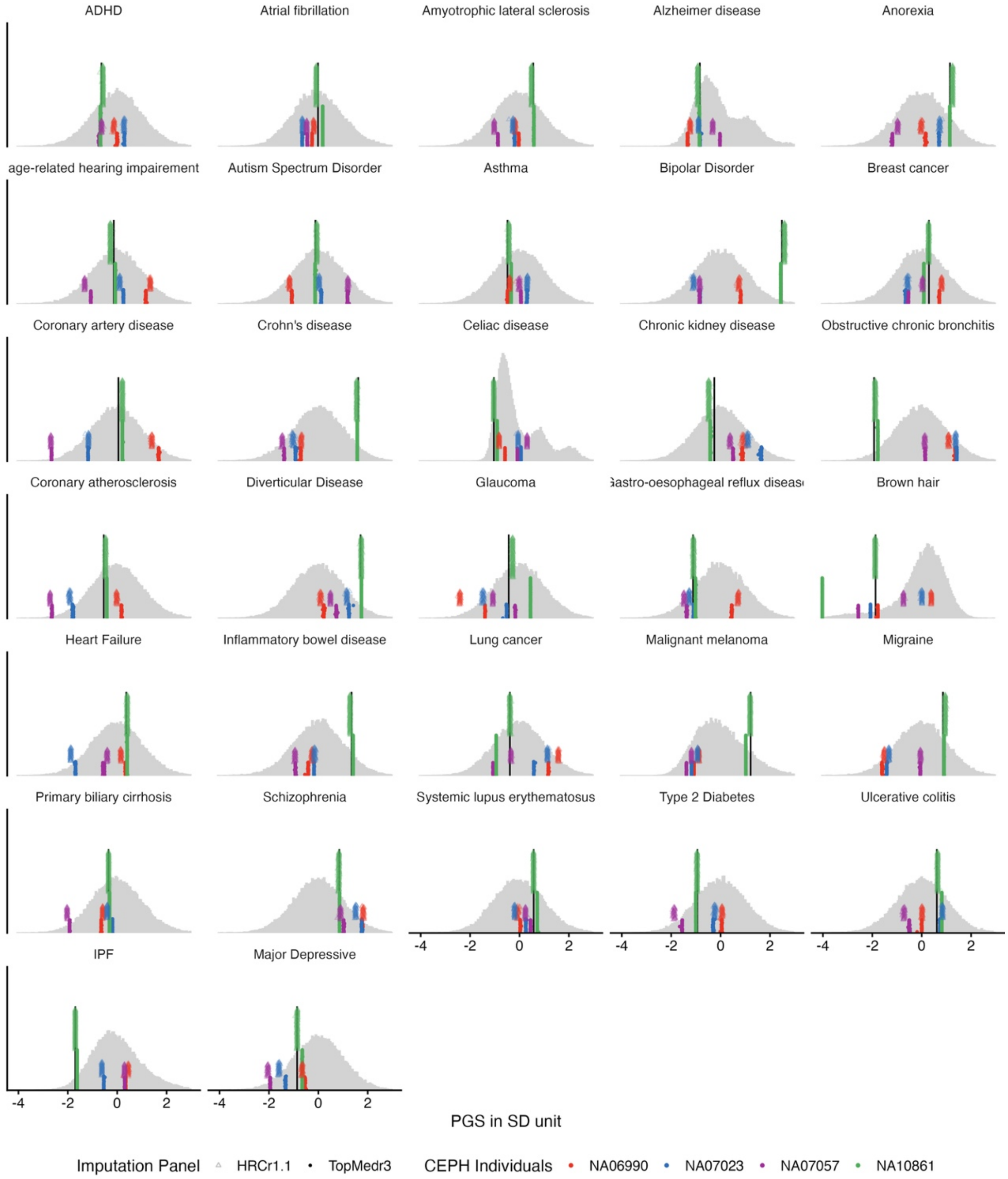

B. 23 quantitative traits collected from different studies.

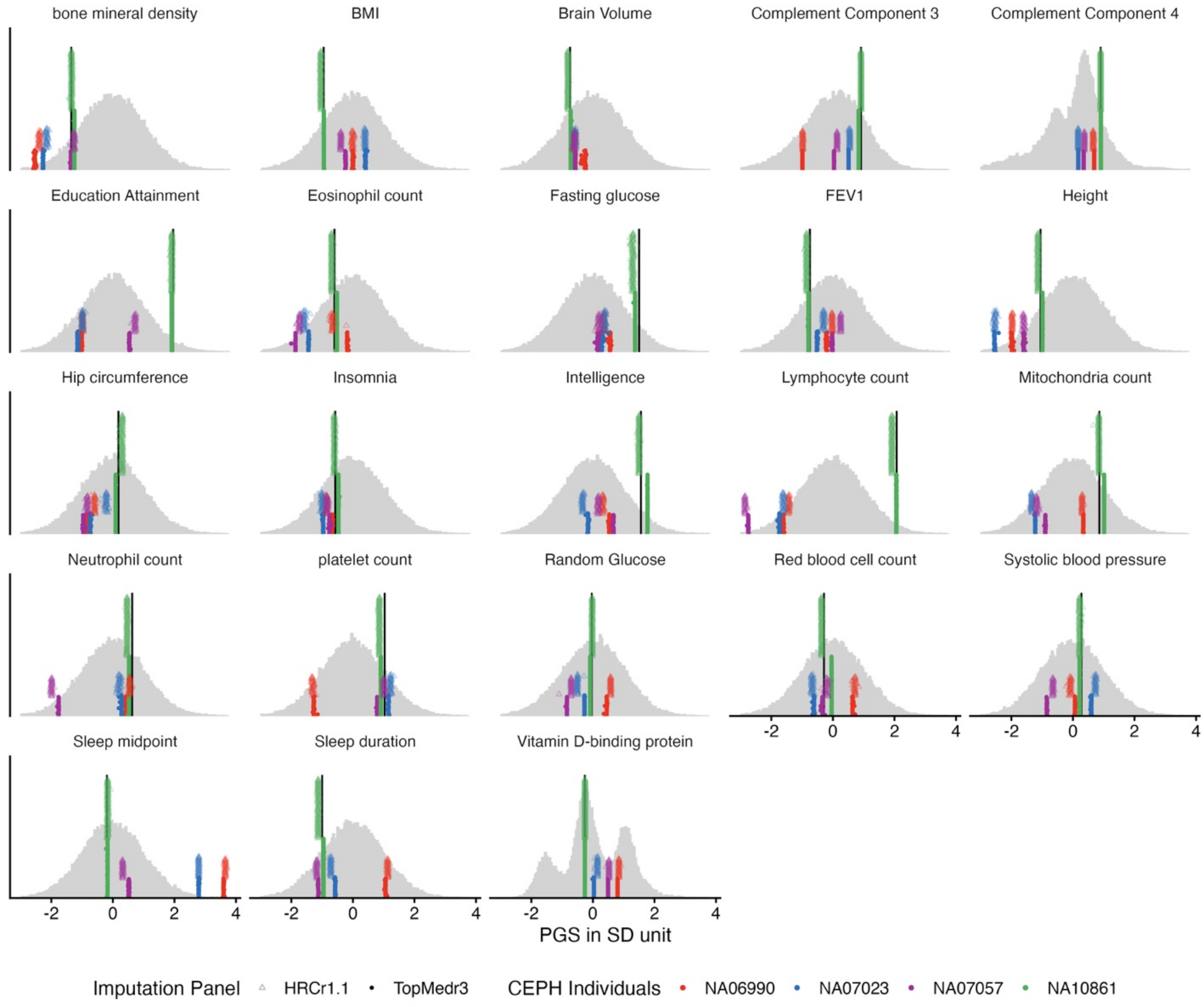

C. 33 traits selected from the 35 urine and blood biomarker in UK Biobank study Sinnott-Armstrong et al., 2021.

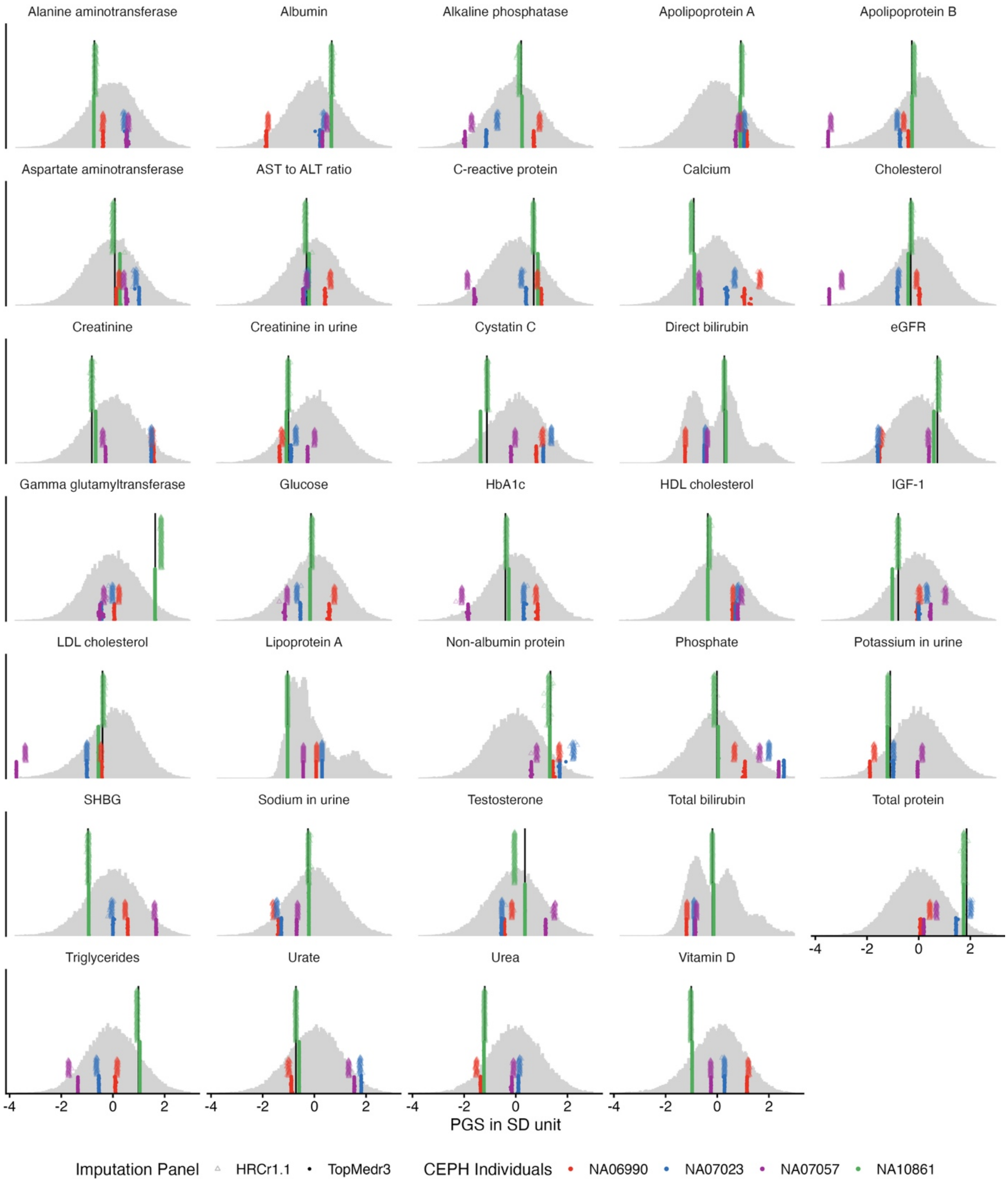

D. 16 Fatty Acid traits from the study Borges et al., 2022.

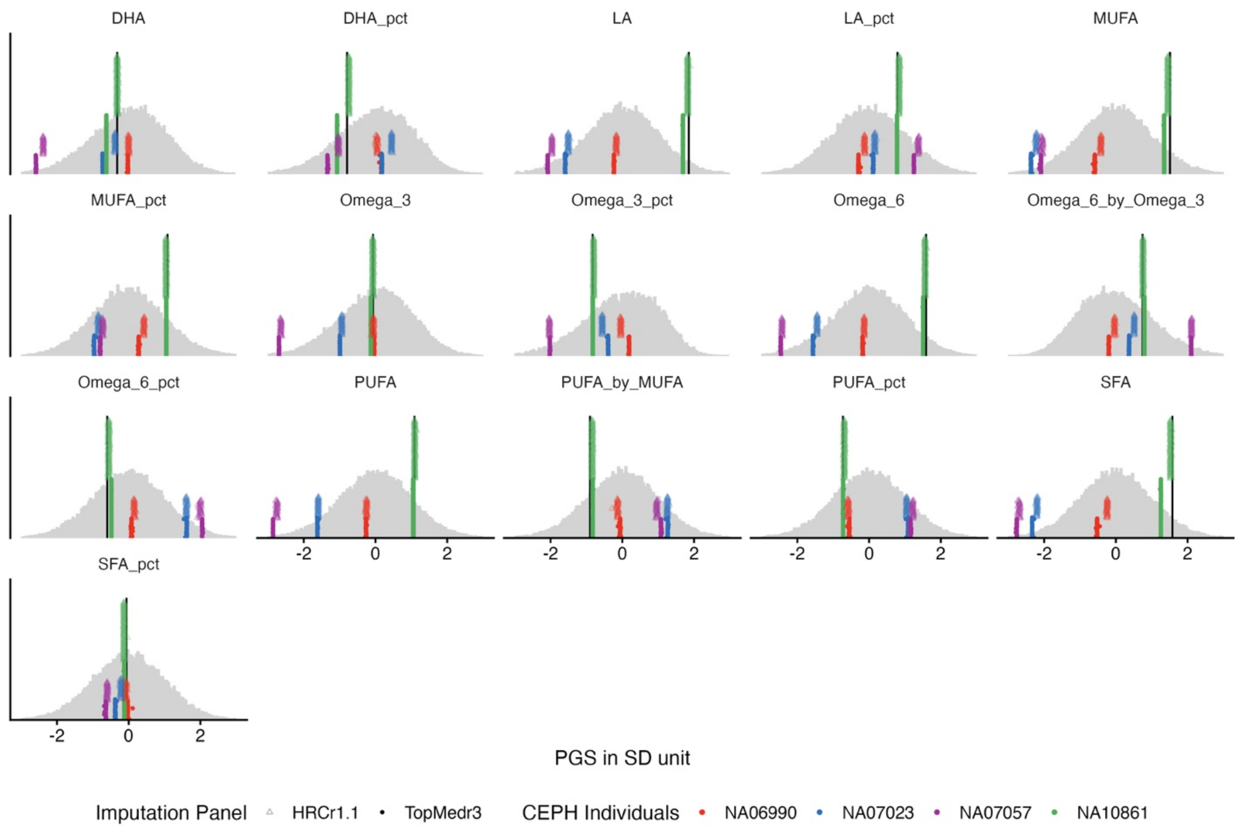

E. 10 Plasma Proteomics related traits from the study Sun et al., 2023

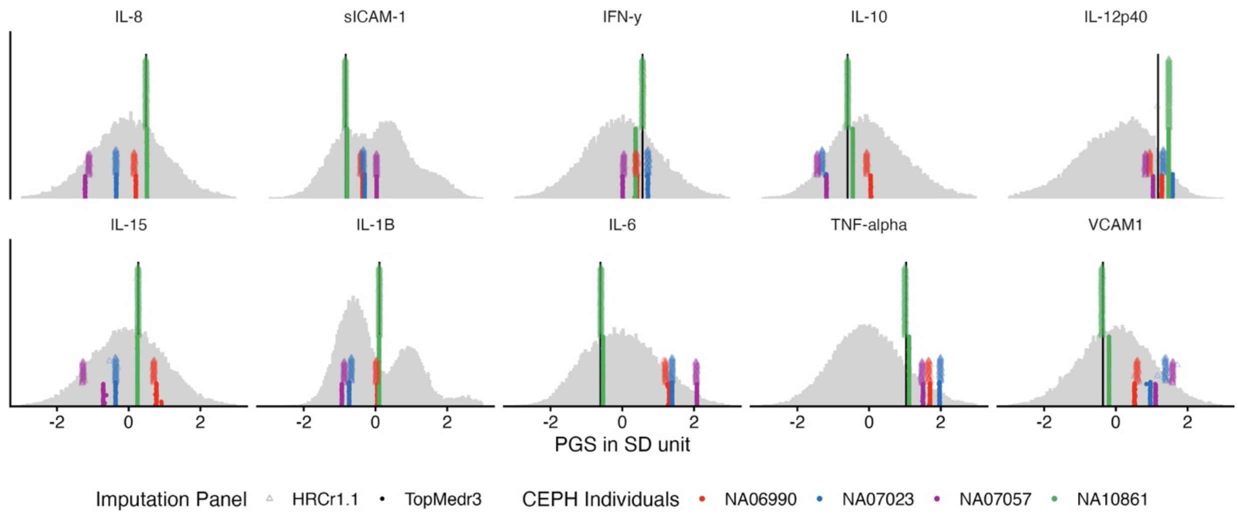

Figure S6. Histogram of the absolute difference between TopMed and HRC imputed PGS across 115 traits and across 4 CEPH individuals processed through QIMRB research pipeline

A. **Using SNPs present in each genotyping/sequencing instance**
B. **Using SNPs present in all genotyping/sequencing instances**

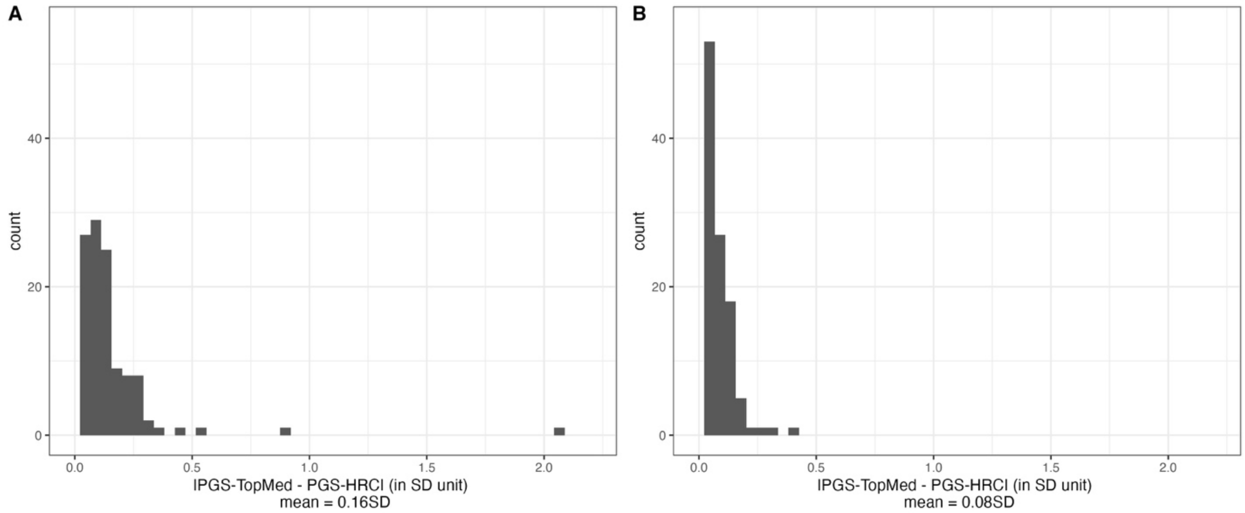

REFERENCES

1. Danecek P, Bonfield JK, Liddle J, Marshall J, Ohan V, Pollard MO, et al. Twelve years of SAMtools and BCFtools. Gigascience. 2021;10(2).
2. Loh PR, Danecek P, Palamara PF, Fuchsberger C, Y AR, H KF, et al. Reference-based phasing using the Haplotype Reference Consortium panel. Nat Genet. 2016;48(11):1443-8.
3. Rubinacci S, Delaneau O, Marchini J. Genotype imputation using the Positional Burrows Wheeler Transform. PLoS Genet. 2020;16(11):e1009049.
4. MSAC_MammaPrint_GeneExpressionProfileTest. http://wwwmsacgovau/internet/msac/publishingnsf/Content/13761-public.
5. McKenna A, Hanna M, Banks E, Sivachenko A, Cibulskis K, Kernytsky A, et al. The Genome Analysis Toolkit: a MapReduce framework for analyzing next-generation DNA sequencing data. Genome Res. 2010;20(9):1297-303.
6. Rubinacci S, Hofmeister RJ, Sousa da Mota B, Delaneau O. Imputation of low-coverage sequencing data from 150,119 UK Biobank genomes. Nat Genet. 2023;55(7):1088-90.
7. Pasaniuc B, Zaitlen N, Shi H, Bhatia G, Gusev A, Pickrell J, et al. Fast and accurate imputation of summary statistics enhances evidence of functional enrichment. Bioinformatics. 2014;30(20):2906-14.
